# Four-year community-wide PM_2.5_ exposure characterization using a low-cost sensor network in a rural valley influenced by residential wood smoke

**DOI:** 10.1016/j.atmosenv.2025.121398

**Published:** 2025-07-18

**Authors:** Nora Traviss, John Stanway, John Woodward, Thomas Webler, George Allen, Mahdi Ahmadi

**Affiliations:** a Northeast States for Coordinated Air Use Management (NESCAUM), Boston, MA, 02111, USA; b Keene State College, Keene, NH, 03435, USA; c University of North Texas, Denton, TX, 76203, USA; d Social and Environmental Research Institute, Greenfield, MA 01301, USA

**Keywords:** Particulate matter, Exposure, Rural, Residential wood heating, Woodsmoke, Low-cost sensors, Purple air, Citizen science

## Abstract

Exposure to fine particulate matter (PM_2.5_) from woodsmoke is a national and global public health concern. While wood heat use is increasing in the Northeast U.S., exposure to woodsmoke in rural valleys in this region remains understudied. Low-cost sensors have recently emerged as a promising strategy to better assess PM_2.5_ exposure in communities across the globe. However, real-world, long-term community air monitoring studies deploying low-cost sensors are lacking. Such studies are necessary to validate performance over time for the goals of assessing exposure trends and providing practical guidance to future community-scale projects. Here, we evaluated PM_2.5_ community-wide exposure over four years in a rural, woodsmoke impacted community that deployed a Purple Air network. We determined significant differences between the PM_2.5_ regulatory reference monitor and Purple Air PM_2.5_ concentrations across the community, over multiple heating seasons, at both hourly (*p* < 0.01) and 8-h average time intervals (*p* < 0.001). PM_2.5_ was especially elevated in the evenings (6 p. m.–2 a.m.), with a maximum 1-h average of 81.7 μg/m^3^ and a maximum 8-h average of 78.7 μg/m^3^. Consistent with other performance evaluations in the literature, we determined Purple Air sensors have low bias after correction (2.4 % Normalized Mean Bias Error [*NMBE*], 3.3 μg/m^3^ Root Mean Square Error [*RMSE*]). Co-located sensors were reliable in harsh winter conditions for up to three years. We suggest quality assurance, data management, correction model selection, and citizen science partnerships are critical considerations for sensor network deployments aiming to assess long-term exposure and health.

## Graphical Abstract

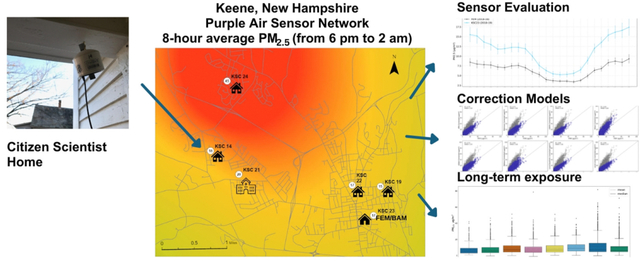

## 1. Introduction

Particulate matter (PM) from woodsmoke is a known public health concern (Avenbuan and Zelikoff, 2020; Naeher et al., 2007; Nassikas et al., 2023). Short term exposure to elevated particulate matter concentrations from all sources is associated with multiple negative cardiopulmonary health effects including ischemic stroke, vascular injury, oxidative stress, systemic inflammation in healthy adults, increased cardiopulmonary mortality (Pope III et al., 2006, Pope III et al., 2016, Pope III et al., 2020) as well as asthma (Mirabelli et al., 2016). In a nationwide spatiotemporal analysis of PM_2.5_ pollution and life expectancy in the USA, very low levels of air pollution – as low as 3 μg/m^3^ – are associated with loss in life expectancy; these losses are larger when considering other factors such as income, education and race (Bennett et al., 2019). There is limited epidemiological research in the U.S. on the long-term health impacts of wood heating in rural areas, even though use of solid fuels such as wood and pellets for heat is common in rural areas, and this population is at higher risk of lung disease such as chronic obstructive pulmonary disease (COPD) (Moore et al., 2019; Nassikas et al., 2023; Paulin et al., 2013; Raju et al., 2019; Rogalsky et al., 2014).

Previous studies from rural valley communities have examined the impact of residential wood heating (RWH) on ambient air quality in western U.S. states. Researchers have investigated rural Libby, Montana, likely the most well-studied U.S. case of woodsmoke-associated PM_2.5_, to examine impacts of a woodstove changeout program. Prior to an extensive woodstove changeout program of approximately 1200 stoves, outdoor wintertime PM_2.5_ concentrations averaged 27.0 μg/m^3^ in 2004/2005 (Bergauff et al., 2009). Initially, researchers determined indoor and outdoor PM_2.5_ decreased by 71 % and 20 %, respectively, by the end of the woodstove changeout program (Ward and Noonan, 2008; Bergauff et al., 2009). Future research by the same group determined that elemental carbon concentrations did not decrease, and some organic acid emissions increased (Ward et al., 2011). Researchers in other U.S. communities impacted by RWH such as Fairbanks, Alaska have reported high outdoor PM_2.5_ concentrations (1-hr max of 89 μg/m^3^, 24-hr max of 40 μg/m^3^, Yang et al., 2024), as well as elevated indoor air PM_2.5_ concentrations (range, 5.7–77.7 μg/m^3^, Ward et al., 2017). The U.S. Environmental Protection Agency (EPA) designated the Fairbanks, AK area as a PM_2.5_ non-attainment area in 2009, and today Fairbanks continues to exceed the PM_2.5_ 24-h average standard of 35 μg/m^3^ (Brune and Ward, 2024).

The Northeast and New England region, an area of the country where homes commonly use RWH in rural areas, remains relatively understudied, though a few indoor air quality studies have been performed (Fleisch et al., 2020; Paulin et al., 2025). Recent analysis in 2022 determined that use of wood for heating is increasing in the Northeast, including the New England states (Marin et al., 2022). Widespread use of wood heating poses unique challenges in rural valley communities in the Northeast prone to temperature inversions, as elevated PM_2.5_ concentrations can remain trapped near the valley floor during cold, calm evenings with little to no wind. Previous mobile air monitoring investigations in the Adirondacks in New York identified communities with elevated woodsmoke PM_2.5_ values that were likely a result of wood heating (Allen et al., 2011, Allen et al., 2020). These areas did not have nearby Federal Reference Monitors (FRM) or Federal Equivalent Monitors (FEM) to assess ambient air quality.

Low-cost sensor technology may be able to address research gaps to characterize long-term PM exposure in rural communities impacted by RWH. Increasingly, low-cost air sensors are being used by citizens all over the world who want to know the air quality in their local communities (Hano et al., 2020; Morawska et al., 2018; Schaefer et al., 2020). In the U.S., low-cost sensors have gained attention due to their ability to fill in spatial gaps in rural areas where regulatory-grade monitors do not exist. Researchers used low-cost sensors to determine detailed spatial-temporal indoor and outdoor PM_2.5_ exposure relationships in homes in an upstate New York region influenced by woodsmoke (Ferro et al., 2022). However, citizen scientists can also participate in collecting data via installation of low-cost PM sensors to determine air pollution in their communities (Kaufman et al., 2017; Schaefer et al., 2020). Purple Air (PA) monitors have become a popular choice for particulate matter monitoring, due to their low-cost, field ruggedness and WiFi connectivity. The company that makes PA sensors also provides a publicly accessible “live” map on its website where anyone can see PM concentration data in near real-time from thousands of instruments across the world. Detailed evaluations of PA monitors have been completed in recent years, though most of these studies have focused on sensor performance (bias) and development of correction equations, as PA readings generally overestimate PM_2.5_ concentrations (Barkjohn et al., 2021; Magi et al., 2020; Malings et al., 2020; Sayahi et al., 2019; Wallace et al., 2021). Most researchers have found that after calibration with local reference monitors, PA accuracy can be improved to a root mean squared error (*RMSE*) and mean absolute error (*MAE*) between 2 and 4 μg/m^3^ with excellent precision between the two sensor channels (Barkjohn et al., 2021; Magi et al., 2020; Malings et al., 2020).

The applications for PAs are many, including hot spot identification, air quality alerts, air monitoring in environmental justice communities, and long-term pollution monitoring. Researchers have analyzed PA performance in different end-uses such as monitoring wildfire smoke (Holder et al., 2020), near-road traffic emissions in an urban setting (Magi et al., 2020), an urban valley with wintertime cold air pools (Sayahi et al., 2019), and a multi-city PA sensor network in Greece to evaluate intra- and inter-city PM_2.5_ variability (Dimitriou et al., 2023). Fewer studies have reported on long-term characterization of community-wide PM_2.5_ exposure. More recently, studies from Southern California have reported on the use of PA sensors to evaluate air quality in disadvantaged communities (Lu et al., 2022), to perform an environmental justice analysis of wildfire impacts (Kramer et al., 2023), and to examine PA real-world performance in an engaged community setting (Connolly et al., 2022). Still, these studies reported data from limited time frames ranging from months to 1.5 years, demonstrating the effectiveness of PAs for acute exposure monitoring, but open questions remain for long term exposure assessment applications.

In this project, we report on the long-term use of PA sensors in a community-wide network established in 2018 in rural southwest New Hampshire (NH). The city of Keene, NH is a valley community long considered an area of concern for woodsmoke by the New Hampshire Department of Environmental Services (NHDES) due to elevated wintertime PM_2.5_ concentrations and 20 % of residents reporting use of solid wood for heating (NHDES, 2012, NHDES, 2018, see Supplemental Information, Appendix A, for more background). Since 2014, students and faculty members at Keene State College (KSC) worked with citizen scientists and local schools on various community air quality monitoring projects culminating in the Keene Clean Air project that deployed PA sensors throughout the city.

While the Keene Clean Air project has a long history with multiple interdisciplinary threads, such as community collaboration, citizen science, and public outreach on proper wood burning, this paper focuses on the following specific project outcomes: 1. Analysis of spatial and temporal variability across the community at the neighborhood scale, comparing corrected PA PM_2.5_ against NHDES FEM PM_2.5_ concentrations; and 2. Long-term assessment of community PM_2.5_ exposure in a rural valley impacted by RWH. Our team of NESCAUM and KSC researchers compared local data driven correction models (Keene data) against the US EPA national correction equation (Barkjohn et al., 2021) and other selected models from the literature. We also discuss the practical implications of this study’s results for future PA community-wide deployments. While low-cost sensors are of increasing interest in environmental justice communities (Kaufman et al., 2017), real world implementation data are needed to resolve the inherent challenges of community air monitoring projects such as leveraging limited resources and expertise, technical management of big data sets, ensuring quality data, and data analyses that are ultimately useful to researchers, government agencies, and the community to guide potential interventions to improve air quality.

## 2. Methods

### 2.1. Study site & Relevant community background

The city of Keene (population 23,000) is approximately 37 square miles located in a wide and shallow glacial valley in rural Cheshire County in southwest New Hampshire. There are no major highways (traffic) or major industrial sources. Keene has a long history of elevated woodsmoke due to temperature inversions on cold, calm evenings with little wind. Details related to the history of RWH impacts in Keene, the various collaborative efforts to improve air quality since 2009, and the Keene Clean Air Project (www.keenecleanair.org) are outlined in Appendix A of Supplemental Information. For this paper, we define the heating season as December 1 to February 28, inclusive. Our study period for PM_2.5_ analyses included four heating seasons for the years 2018/2019, 2019/2020, 2020/2021, and 2021/2022. The only NHDES monitoring station for rural Cheshire County is in downtown Keene, thus Keene is the proxy for the rest of the county for Clean Air Act compliance. Keene has remained an area of interest for the NHDES due to elevated PM_2.5_ from RWH compared to the rest of the state (NHDES, 2021).

We deployed units in distinct residential neighborhoods generally defined as West Keene, East Keene, Keene High School, and North/Central Keene. KSC students or faculty assisted in the placement of the units to ensure they were approximately 1.5–3 m above ground level, not near any vents or chimneys, received good air flow, and had a strong WiFi signal. In 2018/2019, the Keene Clean Air project had deployed about 7–10 PA units across the community. Over the next few years, the number of active units ranged from 4 to 10 at any given time, depending on who joined or left the program. Two PA units (one in 2018/2019) were collocated outdoors under cover next to the NHDES FEM monitoring instrument air intake (Beta Attenuation Monitor [BAM] Met-One Model 1020) near downtown Keene through an ongoing collaboration with NHDES. All PA units underwent initial testing in the Keene State College laboratory prior to deployment in the community.

### 2.2. PA sensor data quality: data cleaning, limit of detection, and correction model comparisons

PA units in this study contained two side by side Plantower sensors (Model 5003) that use scattered light from a laser in a principle of operation closer to an optical particle counter-type sensor (Ouimette et al., 2024), rather than a nephelometer-type sensor as originally hypothesized (Ouimette et al., 2022). The Plantower 5003 sensor sends discrete pulse outputs related to an individual particle’s scattered light, then bins the associated pulse amplitude in different size categories (Ouimette et al., 2024). From there, the Plantower proprietary algorithm calculates PM_1_, PM_2.5_ and PM_10_ mass concentrations (Sayahi et al., 2019; Wallace et al., 2021). Several independent research teams have developed empirical correction equations to adjust for the well-known overestimation of PA PM_2.5_ mass concentration values (from the Plantower algorithm’s CF_1 output) by comparing to measurements from research grade instruments and FEM monitors (Ardon-Dryer et al., 2020; Barkjohn et al., 2021; Holder et al., 2020; Magi et al., 2020; Malings et al., 2020; Sayahi et al., 2019).

Development of correction methods for PA’s PM_2.5_ CF_1 output (the average of the CF_1a and CF_1b channels from each of the two Plantower sensors) remains an active area of research. Therefore, we compared correction methods available in the literature (Table S4) to select the most suitable one based on the bias after correction. Our methods to compare correction models are outlined in detail in Appendix B in Supplemental Information. We downloaded the average concentration data for both Plantower sensors (CF_1a and CF_b) at 2-min time resolution, which equates to thirty 2-min readings per hour. For all CF_1 a and b concentration data, we adapted and applied the following data cleaning procedures described in Table 1 in Connolly et al. (2022), which follows the steps outlined in Barkjohn et al. (2021), briefly summarized here: we excluded 2-min data if the CF_1 a or b channel differed by more than 10 μg/m^3^, if the difference between the channels exceeded 5 μg/m^3^ and 61 %, or if any negative PA values were reported. There were no values in the study that ever exceeded 100 μg/m^3^. We determined a limit of detection (LOD) for all PA sensors in the study according to the method presented by Wallace et al. (2010, 2021) where the LOD is defined as the lowest mean value of the two PA channels (*μ*) at which more than 95 % of the mean values have a signal-to-noise ratio (*μ/σ*) greater than 3, where *σ* is the standard deviation of the channel a and b outputs.

### 2.3. Spatial and temporal variability of PM_2.5_ concentrations across heating seasons

We plotted time series analyses of the uncorrected PA PM_CF_1 and reference BAM PM_2.5_ concentrations (1-h averages) to check for diurnal patterns. Scatterplots also assessed temporal variability for collocated units across all heating seasons. We next corrected the data using the Barkjohn et al. (2021) national correction equation. Then, to evaluate spatial variability, we developed a map with the ArcPy extensions to ArcGIS PRO 3.2.1. We used the same Python program that displays the “live” map of PM_2.5_ concentrations on the Keene Clean Air project website (www.keenecleanair.org), with the following adjustments. For the paper, instead of “real-time” snapshots as displayed on the website, we created maps for the evening periods (from 6 p.m. to 2 a.m., 8-h average) during the dates December 8, 2018 to December 10, 2018 and the evening of December 17, 2018. We selected these specific dates as exploratory data analysis showed an extended air inversion took place during this time.

On the maps, we interpolated the 8-h average concentrations across Keene using simple kriging. We plotted the resulting values on a color ramp from green (lowest concentration) to red (highest concentration) showing the spatial concentration coverage of the area surrounding the sensors. The map can be recreated for any date in the study, and the dates selected here are simply illustrative of the application principles during an inversion event. Color gradient changes are based on proximity of each point to the nearest PA unit, so if there is no unit in a neighborhood, the nearest units might be too far away to show a semi-quantitative estimate by color. The map provides a simple visualization of the 8-h average concentration at the actively reporting PA unit, and then semi-quantitative, estimated concentration gradients centered around the units.

To assess significant differences in corrected PA concentrations in neighborhoods across the community compared to the downtown BAM reference instrument, we performed one-way Kruskal Wallace ANOVA with Dunn’s post-hoc test on 8-h average concentrations (10 a.m.–6 p. m., 6 p.m. to 2 a.m., 2 a.m.–10 a.m.) for all PAs in each heating season that passed quality criteria for data cleaning. Previous mobile monitoring had indicated elevated PM_2.5_ occurs in the evening hours after people return home and start their woodstoves (Fig. S4).

### 2.4. Long-term community PM_2.5_ exposure

Only PA 1-h PM_2.5_ concentration data that passed the previous section’s QA/QC criteria participated in the exposure data analysis. After the comparison study (methods described in Appendix B, Supplemental Information, results in section 3.1), we selected the EPA national correction equation (Barkjohn et al., 2021) for the exposure analysis for the following reasons. In the exploratory data analysis phase, we observed in the 2021/2022 season several high concentration outliers in the data for both the BAM and PA. Our investigation revealed a serious fire occurred in downtown Keene near the BAM, which destroyed multiple buildings and attracted media attention on January 8 to 9, 2022 (WMUR, 2022). Removing these high PM_2.5_ outliers substantially improved our local correction models. However, as a result, we noticed our locally derived models did not perform well at concentrations above 35 μg/m^3^ because our collocated dataset now had a relatively narrow range (all concentrations less than 70 μg/m^3^). A 1-h PA concentration of >35 μg/m^3^ was not unreasonable to expect in some of the more densely populated neighborhoods in Keene based on visual observation, previous mobile monitoring (Fig. S4), and our exploratory data analysis. Additionally, after plotting time-series analyses, we observed problematic issues with one collocated sensor (KSC-11) during the 2020/2021 season. We further explain our rationale for selecting the EPA model over others in the results and discussion section. We developed boxplots to display the average hourly PM_2.5_ concentrations, and 8-h average concentrations, across the Keene community for the four heating seasons.

## 3. Results and discussion

### 3.1. PA sensor data quality: data cleaning, limit of detection, and correction model comparisons

Overall, the 15 total PA units in this study performed well in winter, demonstrating field ruggedness and reliability. In each season, most PAs reported less than 2 % of the hourly data not meeting QA/QC due to differences between the CF_1 a vs b channel at the 2-min time interval. There were four sensors in the 4-year study with 2–12 % of 2-min data not meeting the CF_1 a vs. b criteria in one season, the most notable being sensor KSC-11 (9.2 % data excluded) in 2019/2020, as this sensor was collocated with the BAM. Most PAs reported no issues with the humidity and temperature checks, though three sensors excluded 3–4.8 % of their 2-min dataset in one season due to this cleaning step. There were sensors that did not meet 80 % data completeness criteria due to outages (no data reporting from the WiFi), most notably KSC-03 and KSC-01 in 2020/2021. Losing the internet connection was the main reason a PA unit reported low data completeness, similar to the reports from a community study at UCLA (Connolly et al., 2022). Our overall QA/QC results were comparable or better than reported by Barkjohn et al. (2021), who evaluated 50 sensors across multiple states. A complete list of PA units deployed per heating season for more than 4 weeks, their locations, and their distance from the BAM is in the Supplemental Information (Table S2).

We evaluated eight models that determined relationships between the uncorrected hourly PM_CF_1 data and the hourly BAM data: two models developed by our team (Appendix B, SI); five correction models developed by other research teams (Table S4); and one model developed by Wallace (2023) using the Keene particle number concentration data instead of PM_CF_1. Details of our approach are described in Appendix B, Supplemental Information.

**Fig. 1.**
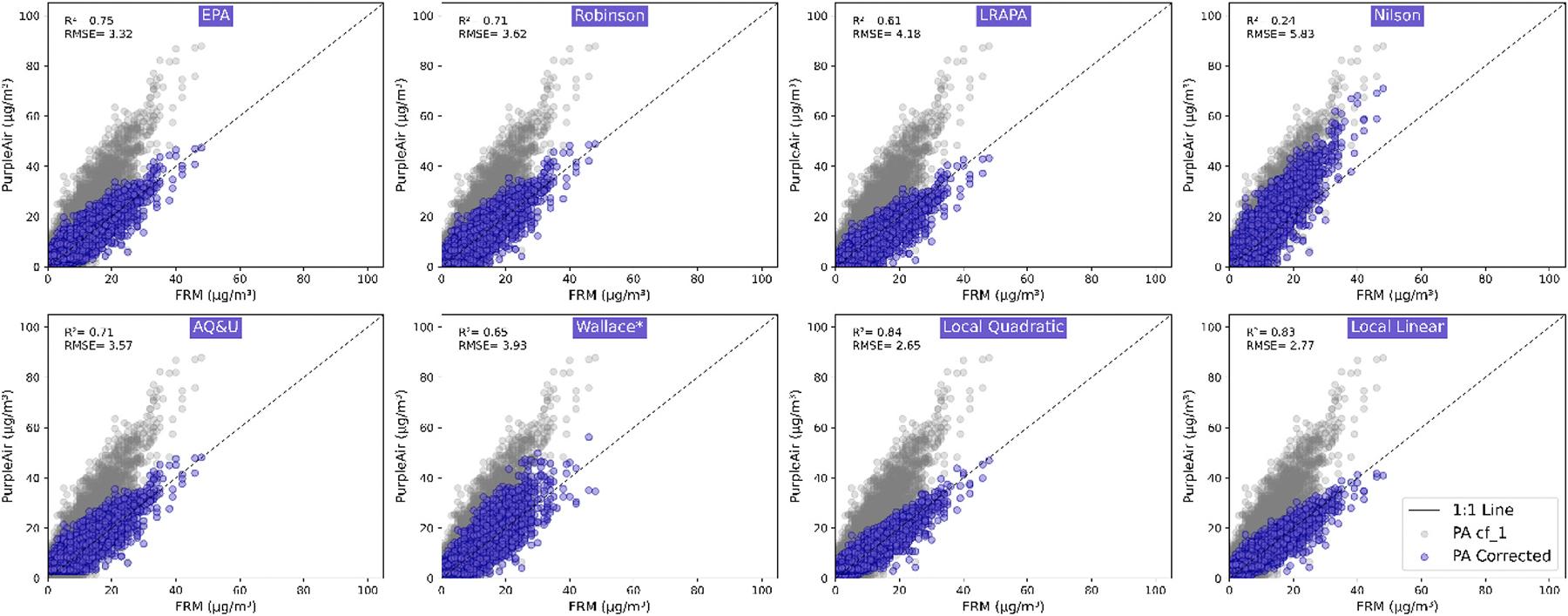
Comparison of BAM data versus uncorrected hourly PM_2.5_ (PM_CF_1) (gray dots) and corrected PM_2.5_ (purple dots) for each of eight models (local and Table S4). Wallace* has an asterisk as we used local particle number concentration data to derive a local correction factor (CF) per Wallace et al., (2021), 2023, resulting in a higher, local CF of 4.8 compared to 3.4 CF in Wallace (2023), Eq (7). (For interpretation of the references to color in this figure legend, the reader is referred to the Web version of this article.)

**Fig. 2.**
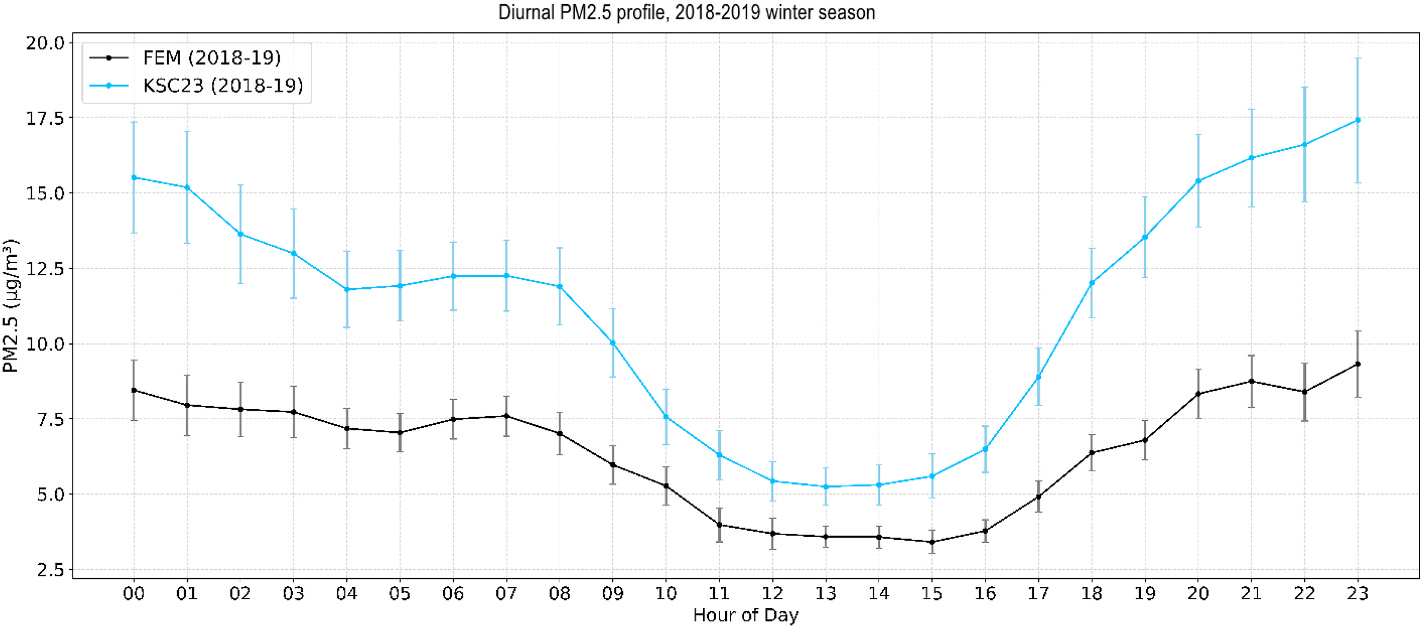
Heating season 2018/2019 time series analysis of 1-h average BAM and collocated PA PM_2.5_ concentrations (with standard error bars).

The PA’s uncorrected PM_CF_1 data (Fig. 1) overestimated the PM_2.5_ mass concentration as reported by the BAM instrument, as observed by others (Holder et al., 2020; Magi et al., 2020; Malings et al., 2020). After correction, the best models in Fig. 2 included the two local data models (Local Quadratic, *r*^2^ = 0.84, *δ*_*y, rms*_ (root-mean-square-deviation, hereafter referred to as *RMSE*) = 2.65 and MLR, *r*^2^ = 0.83, *RMSE* = 2.77) and the next best was the EPA (*r*^2^ = 0.75, *RMSE* = 3.32).

The overestimation of uncorrected PM_CF_1 data and the improvement post-correction were further illustrated in Table 1, which shows the normalized mean bias error (*NMBE*) of each model for the full dataset and at the different concentration brackets that align with the EPA Air Quality Index (AQI) platform. Without correction, the PA PM_CF_1 overestimated PM_2.5_ concentrations by 37–78 %. While all models reduced the bias compared to the uncorrected PM_CF_1, the EPA national correction model performed the best across the higher concentrations (>35.5 μg/m^3^) expected in Keene for community exposure, based on our exploratory data analysis of the entire (uncorrected) PA dataset. The Wallace correction had better *NMBE* compared to the EPA correction in the “good” range of 0–9 μg/m^3^ (3.9 %) and the moderate range of 9.1–35.4 μg/m^3^ (2.5 %). However, the EPA correction performed the best of all models in the “unhealthy” range over 35.5 μg/m^3^ (*NMBE*, 3.4 %). The EPA correction also had overall lower normalized root mean square error (*NRMSE*) compared to other models in the literature, and comparable *NRMSE* (26 % and 13 %) to the local data driven models in the “moderate” and “unhealthy” range (Table S1).

The increased bias in the locally derived models in the range >35.5 μg/m^3^ is likely due to relatively few data points observed at the BAM reference in this range during our study (n =13), whereas the dataset for the EPA correction equation is much larger, which can account for larger variation in a dataset (Barkjohn et al., 2021). The lower bias (and lower *NRMSE*, 13 %, Table S1) of the EPA model in the >35 μg/m^3^ concentration range was a key consideration to select the EPA correction for the community exposure analysis. However, we also considered the practical implications of selection of a less-computationally intensive model like the EPA correction for smaller communities and states potentially without access to statistical expertise. We suggest research groups with statistical expertise also consider local data driven correction models, and the Wallace model based on particle counts, to improve the accuracy of the exposure analysis, in addition to the EPA model.

We determined low limits of detection well under 2 μg/m^3^ for 13 out of 15 PA units in the study, except for KSC-04 at 3.2 μg/m^3^ and KSC-21 at 6.1 μg/m^3^ (Table S3). The average LOD including all the units in the study was 1.2 μg/m^3^. Wallace et al. (2021) calculated LODs of 1.77 μg/m^3^ and 2.55 μg/m^3^ at two different locations in California using the PA CF_1 channel data but determined lower LODs using their particle number concentration (ALT) method. The authors suggest that since many regions in the U.S. may have a large percentage of low ambient PM measurements near zero, the lower LOD is another key benefit of the ALT model (Wallace et al., 2021). Other researchers have taken different technical approaches to PA LOD determination with varying results: Magi et al. (2020) estimated a PA LOD of 5 μg/m^3^, and Sayahi et al., 2019 reported 2.62–3.00 μg/m^3^ in the winter for comparable Plantower 5003 sensors. Sources of pollution (urban traffic, woodsmoke, industrial) and the expected range (low to high) of ambient concentrations are important considerations in the selection of low-cost sensors.

We recommend governmental agencies and community monitoring groups consider their goals for data quality carefully before building a sensor network. If the goal is to raise public awareness of local air quality, then precision and bias goals of up to 50 % may be adequate (EPA, 2019). EPA (2019) recommends reducing bias and precision to under 30 % (or as low as possible) if understanding exposure trends for epidemiological or health studies is the goal. Implementing the QA/QC steps followed in this study and applying several of the correction models from Table 1 would also improve the overall accuracy of results to less than 20 % bias, but these methods have varying degrees of computational demand, which may be a challenge for community-run programs. For this work, we ultimately selected the EPA correction by Barkjohn et al. (2021).

We also suggest performing frequent checks of data using QA/QC steps to identify problematic sensor performance, and periodic local corrections on a per season or annual basis to meet project data quality objectives. We recommend plotting uncorrected PA data via time series analysis over long time periods as a simple way to check sensor performance in the field. We discuss this further in the next section. The EPA Enhanced Sensor Guidebook (2022) reviews several other practical strategies for collocation of sensors and corrections but also acknowledges these strategies (such as moving sensors from the field to an FEM reference monitor or an FEM to the field) can be impractical at times or highly expensive.

### 3.2. Spatial and temporal variability of PM_2.5_ concentrations across heating seasons

#### 3.2.1. Time series analysis

Fig. 2 shows the 1-h average PM concentration for each hour during the 2018/2019 season for the PA unit (KSC-23) co-located with the BAM (reference monitor). The results for the other seasons are in the Supporting Information (Figures S11 a, b, and c). The PA unit generally tracked the BAM but with a ~50 % positive bias. The diurnal pattern of higher concentrations in the evening was expected with RWH being the main source of PM in the community. The lowest concentrations of PM (<5 μg/m^3^) were in the afternoon (noon until 5 p.m.) while most people are at work or school, and the concentrations climbed after 5 p.m. when residents returned home and started their woodstoves. PM_2.5_ peaked during the evening during the 9–11 p.m. hours. Vertical mixing is likely also changing throughout the day, with less mixing in the late evening to early morning hours. These overall temporal trends held over all heating seasons in the study. The BAM and PA somewhat slightly diverged as concentrations rise in the evening in 2018/2019, but generally the ratio of ~2.0 (PM_CF_1/BAM) holds over both the day and evening hours.

The principle of operation of the BAM is based on measurement of the attenuation of a low-level beta radiation source by particles collected on a filter tape. The BAM is noisy at the 1-h time interval and has a relatively high LOD of 4.8 μg/m^3^ for an FEM (BAM 1020 Operating Manual). Yet, *r*^2^ values from the local data driven correction models (*r*^2^ = 0.83 to 0.84) were better or comparable to other types of FEM collocation studies (including BAM FEM) in the literature (Magi et al., 2020, *r*^2^ = 0.57 [BAM], Malings et al., 2020, *r*^2^ = 0.53 [BAM], Sayahi et al., 2019, *r*^2^ = 0.89 [TEOM]). Based on evaluation of the time series plots (Fig. S11 and Appendix B), we noted performance issues and replaced both co-located PAs (KSC-11 and KSC-23) before the 2021/2022 heating season with new PA units for collocation with the BAM. We suggest that good practice for community monitoring projects should include collocating two PA units near an FEM monitor throughout the project duration and plotting time series data periodically to check if sensors need repair or replacement. Doing periodic multiple collocations of all deployed sensors next to FEM monitors is also recommended by EPA (2022) but may not be feasible or practical for community or citizen science networks. Researchers have suggested that collocating within 0.5 km of an FEM is sufficient for comparison purposes (Wallace and Zhao, 2023). Ultimately, we found that certain co-located PAs in this study provided reliable data for up to 3 years.

Our results highlight additional practical considerations for community groups and governmental agencies. Researchers generally perform data analysis after all data is collected. Community groups and government agencies need accurate data in near real-time contexts for ongoing maintenance of the sensor network, air quality decision-making, and public health risk communication. These stakeholders require accurate, quality assured data on a shorter time scale due to decision urgency related to broader air quality improvement goals. We already mentioned keeping two collocated PA sensors near the reference monitor to prevent loss of data integrity in case one is found to be poor performing and plotting frequent time series (of 1-h averages) comparing the PA units’ performance versus the reference. Assessing temporal patterns can also be helpful in identifying sources such as RWH or traffic. Another “lesson learned” as part of the sensor network operation: we developed a Python script that sent faculty and student researchers an automated 24-h error report in the form of a daily email to help track the performance of all PAs throughout the city. The script checked all active sensors and reported those sensors that had performance issues, such as: being offline for more than 30 min, any difference between the a and b channels >10 μg/m^3^, or any CF_1a or b concentration reported greater than 250 μg/m^3^. This resulted in more timely identification of issues and repairs to address the problems. Our review of error report logs determined the most common issue for citizen scientist PAs was being offline due to WiFi connectivity issues or inadvertently shutting off power to the sensor, similar to the issues reported in Connolly et al. (2022).

#### 3.2.2. Spatial variability

**Fig. 3.**
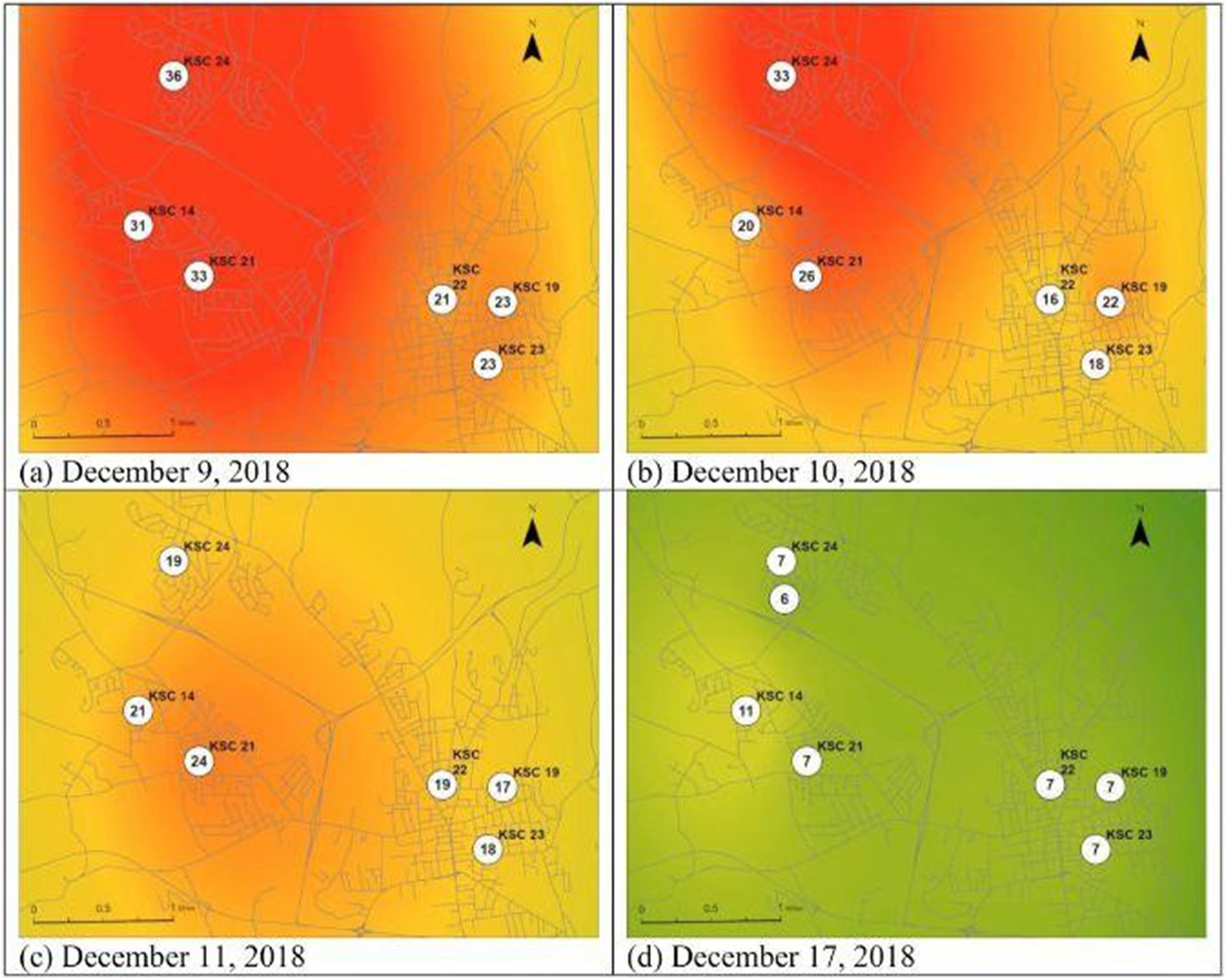
a, b, c, d, from top left: Keene, NH heatmaps using a color ramp from green to red showing 8-h evening concentrations of PM_2.5_ for the dates shown. The PA 8-h concentrations (corrected via the EPA model) are quantified in the white circles. Color gradients here are semi-quantitative and based on distance between the PAs that passed QA/QC. Areas with a higher intensity red coloration have elevated PM_2.5_ levels near the sensor. (For interpretation of the references to color in this figure legend, the reader is referred to the Web version of this article.)

One of the overarching research questions of the Keene Clean Air project was whether the centrally located BAM adequately captured potential spatial heterogeneity in the valley. We plotted spatial maps using the 8-h average PM_2.5_ concentrations (for the 6 p.m. to 2 a.m. period) during the evenings of December 9, 10, 11, and 17, 2018. These maps are shown in Fig. 3 panels a, b, c, and d. KSC-23 was located adjacent to the BAM reference monitor in downtown Keene in the lower right corner of each panel. The distance from KSC-23/BAM to KSC-24 in West Keene is about 5 km. West Keene is a densely populated housing development of single-family homes with approximately 0.25 acres of land per home (Table S2). The distance between KSC-23/BAM and North/Central Keene (KSC-22) and East Keene (KSC-19) is ~1000 m and 800 m respectively in separate neighborhoods, each with a mix of multi-family and single-family housing.

The maps are semi-quantitative in their representations of concentration gradients but highlight two important observations that we identified over multiple seasons in the study. We observed spatial variation across the community on the neighborhood scale, with the West Keene housing development reporting the highest evening 8-h average PM levels in Keene, up to 2 times higher than other neighborhoods. In fact, the maximum hourly PA concentrations across seasons 2018/2019 (71.0 μg/m^3^), 2019/2020 (71.9 μg/m^3^), and 2020/2021 (56.1 μg/m^3^) were from PA sensors in West Keene. Our team had earlier identified this neighborhood as a “hot spot” for further interventions based upon woodpile counts, confirmed by early mapping in 2018/2019, so we implemented targeted outreach in this area using door flyers highlighting EPA Burn Wise principles of “Burn the Right Wood, in the Right Stove, in the Right Way” during that season.

The second major observation is the overall consistently elevated concentration throughout the valley from December 9 to 11, 2018 until the evening of December 17, 2018. This suggests that an ongoing temperature inversion event may have occurred and prevented vertical mixing of the woodsmoke over an extended period. By the evening of December 17, 2018 (from 6 p.m. to 2 a.m. on December 18), this inversion pattern was broken. A close inspection of the time series plot in Fig. S5 during this time, as well as review of the historical meteorology for those dates (cold evenings, little to no wind speed, no snow or rain), support an assessment of an extended temperature inversion that trapped PM in the valley.

Finally, we analyzed spatial variability by ANOVA, comparing PA 1-h and 8-h averages versus the BAM (reference monitor) each season. The ANOVA results are reviewed in Section 3.3, as part of evaluating long-term community exposure.

### 3.3. Long-term community PM_2.5_ exposure

#### 3.3.1. 1-h average PM_2.5_ concentrations

**Fig. 4.**
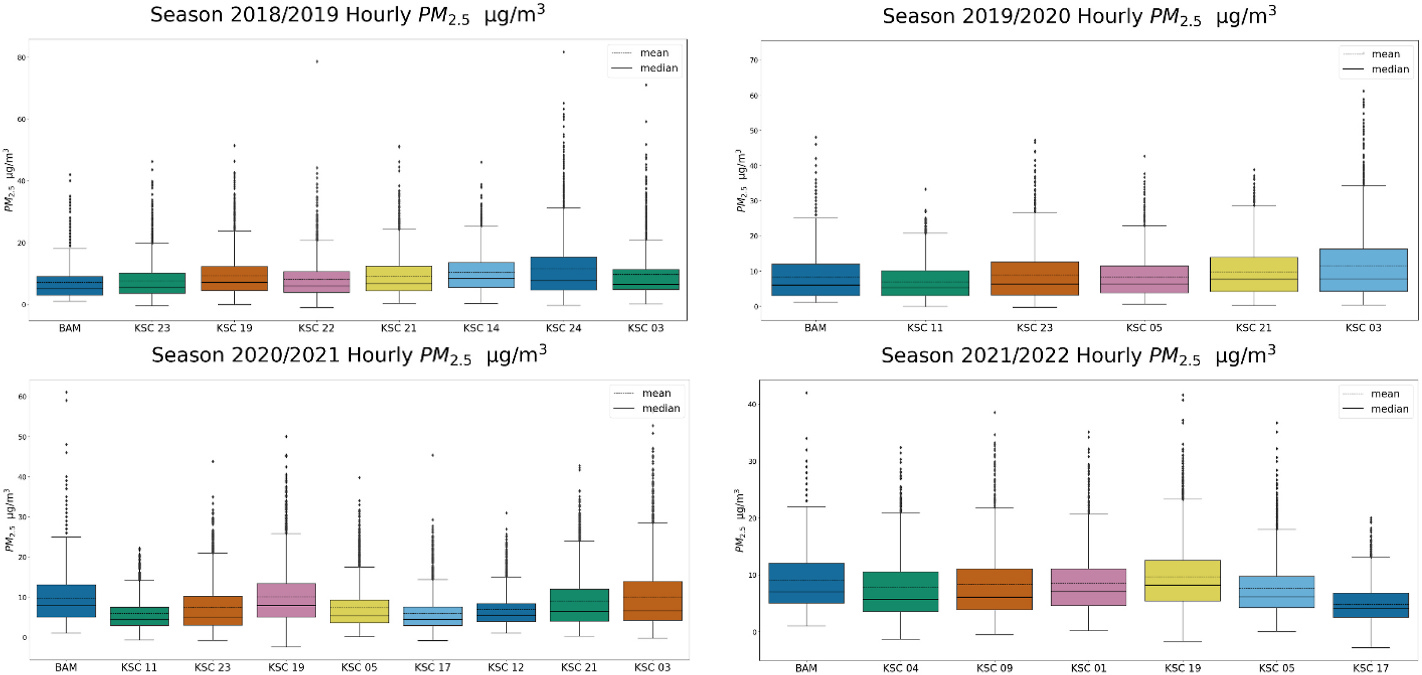
a, b, c, d, from top left: Boxplots of 1-h intervals for PM_2.5_ measurements (in μg/m^3^) in the heating seasons in Keene, NH from the year 2018–2022, plotted in order left to right of increasing distance away from the BAM (0–5000 m). The mean is the dotted line and the median is the solid line. The FEM BAM PM_2.5_ concentration is included for comparison, with KSC-23 being the collocated unit for 2018/2019 season, KSC-23 and KSC-11 being the collocated units for season 2019/2020 and season 2020/2021, and KSC-04 and KSC-09 the collocated units for season 2021/2022. Locations and distances from the BAM are listed in Table S2.

We plotted 1-h boxplots (corrected with the EPA equation, Table S4) per season in Fig. 4. The BAM is the first boxplot in blue on the left, and then every other boxplot is in increasing distance from the BAM. The West Keene units (KSC-24 and KSC-03) represent the furthest distance away from the BAM for 2018/2019, about 5 km. If we look at only the BAM’s hourly PM_2.5_ concentration for each season, heating season 2020/2021 ranked first for exposure (mean 9.7 μg/m^3^, median − 8.0 μg/m^3^, and max − 61.0 μg/m^3^). If we evaluate exposure using PA concentrations across the community, then season 2018/2019 and season 2019/2020 report the highest 1-h PM concentrations, all from the same location at the densely populated single-family neighborhood in West Keene (KSC-24 mean − 11.5 μg/m^3^, median − 7.6 μg/m^3^, max − 81.7 μg/m^3^ [2018/2019], KSC-03 − 11.6 μg/m^3^, median, 7.8 μg/m^3^, max − 71.9 μg/m^3^ [2019/2020]).

In fact, the West Keene units’ 1-h average concentrations were significantly higher than the BAM (*p* < 0.001) in 2018/2019 (7.1 BAM vs. 11.5 μg/m^3^ for KSC-24, 9.7 μg/m^3^ for KSC-03). KSC-03 was also significantly higher than the BAM (*p* < 0.001) in 2019/2020 (8.3 BAM vs. 11.6 μg/m^3^ KSC-03) and 2020/2021 (9.7 BAM vs. 10.0 μg/m^3^ KSC-03). The West Keene PA units reported 2–4 % of their 1-h PM_2.5_ concentrations as > 35 μg/m^3^ from 2018/2019 to 2020/2021. Unfortunately, KSC-03 did not meet QA/QC criteria for data completeness in 2021/2022 (the unit was offline for an extended period) and so 2021/2022 data for this neighborhood was not included in our study. Other neighborhoods in Keene also reported significantly higher hourly PM_2.5_ compared to the BAM (*p* < 0.001, unless noted), including KSC-14 (neighborhood near Keene High School, 2018/2019), KSC-21 (Keene High School, 2019/2020 and 2020/2021), KSC-19 (East Keene, 2021/2022), and KSC-05 (East Keene, 2019/2020 through 2021/2022, p < 0.02). These significant ANOVA results support the existence of spatial variability between the BAM and PA units across Keene, likely due to local variation in woodsmoke sources at the neighborhood scale.

We next discuss an important outlier PA in our study, KSC-17, which was sited at a home in East Keene at an elevation of 780 feet, adjacent to Beech Hill (Fig. S2, red X). We wished to explore a hypothesis from drone studies and visual observation (Fig. S3) that inversions in Keene (elevation ~480 feet at the BAM reference monitor) were shallow, trapping woodsmoke close to the ground. PM_2.5_ hourly average concentrations at only 300 feet above ground level (KSC-17) were significantly lower (*p* < 0.001) than the BAM in the seasons it operated (5.9 vs. 9.7 μg/m^3^ BAM, 2020/2021 to 4.8 vs. 9.0 μg/m^3^ BAM, 2021/2022). This evidence suggests woodsmoke may closely “hug” the cold ground in the winter in Keene, accumulating in the valley’s low-lying areas, such that even being at slightly higher elevation (~300 feet from ground level) can significantly reduce exposure to PM_2.5_.

#### 3.3.2. 8-h average PM_2.5_ concentrations (evening period, 6 p.m. to 2 a.m)

The 1-h averages may be considered a more “acute” exposure. For this reason, we calculated 8-h averages during the evening hours (6 p. m.–2 a.m.) to better understand long-term exposure during the period of prime RWH operation and compared these values against the same 8-h interval for the BAM in our ANOVA. Similar to the 1-h results versus the BAM, the West Keene units KSC-24 (8.72 BAM vs. 15.5 μg/m^3^ [2018/2019]) and KSC-03 (10.2 vs. 15.7 μg/m^3^ [2019/2020] and 11.7 vs. 12.8 μg/m^3^ [2020/2021]) had significantly higher 8-h exposure concentrations (*p* < 0.01) and the overall highest PM exposures in the study. Units KSC-24 and KSC-03 in the West Keene neighborhood reported 3.7–8.6 % of their 8-h PM_2.5_ concentrations greater than the EPA 24-h limit of 35 μg/m^3^ from 2018/2019 to 2020/2021. We also found significantly elevated 8-h average exposure in other neighborhoods across Keene compared to the BAM (*p* < 0.001), including KSC-21 (Keene High School, 2018/2019, 2019/2020), KSC-19 (East Keene, 2018/2019), KSC-14 (neighborhood near Keene High School, 2018/2019), KSC-05 (East Keene, 2021/2022), and KSC-12 (North/Central Keene, 2021/2021). Finally, when evaluating maximum 8-h averages across the seasons, West Keene PAs reported up to 71.0 μg/m^3^ (KSC-3, 2018/2019) but a unit near downtown (KSC-22) the same season also reported a maximum of 78.7 μg/m^3^. The PA at the higher altitude East Keene home (KSC-17), was an outlier for low concentrations in the study, with 8-h averages significantly lower than the BAM (*p* < 0.01).

While the 2021/2022 season appears to have lower overall 8-h averages in the evenings, there were no West Keene sensors that passed QA/QC for data completeness, so it is unclear if air quality actually improved overall across the community, especially as the BAM reported its second highest seasonal 8-h evening average at 10.7 μg/m^3^ during this period (Table S6). Subsequently, in the fall of 2022, there was a period of BAM maintenance and more critically, disconnection of the WiFi for the collocated PA units (KSC-04 and KSC-09) as the NHDES began to transition to a new FEM (Teledyne T640) and new communications systems in the spring of 2023. Due to these limitations, we could not include 2022/2023 heating season data in our study. We include these points to show that maintenance of a sensor network can be challenging due to multiple factors.

#### 3.3.3. Comparison to other U.S. Residential wood heating impacted communities

We next evaluated the Keene PM exposures compared to other RWH community studies in the U.S. Bergauff et al. (2009) determined a post-changeout heating season average of 21.8 μg/m^3^ (2007/2008, based on 24-h averages, heating season defined as November 1 to March 1) in Libby, Montana. This study was based in a rural valley community that participated in a comprehensive woodstove changeout program that replaced over 95 % of uncertified stoves with new woodstoves or other heat appliances (oil or gas). Walker et al. (2021) focused on indoor air quality of homes with RWH but reported time aligned average (over 6 days) ambient PM_2.5_ concentrations of 4.6 μg/m^3^ (Alaska), 1.6 μg/m^3^ (Navajo Nation) and 4.4 μg/m^3^ (western Montana). Yang et al. (2024) reported an outdoor mean of 12.8 μg/m^3^ averaged over their entire study (~5 weeks), with a range of 24-h averages from <5 to ~40 μg/m^3^ in Fairbanks, Alaska (a community impacted by RWH). For Fairbanks, the 2021–2023 PM_2.5_ design value (used by EPA to designate nonattainment areas and defined as the three-year average of the 98th percentile of 24-h PM_2.5_ concentrations) is 67 μg/m^3^, showing high levels of PM_2.5_ in this region due to woodsmoke and unique wintertime meteorology (Brune and Ward, 2022; U.S. EPA, 2024).

Most of these studies in RWH impacted communities did not examine 8-h evening PM_2.5_ concentrations, where elevated exposure to PM from RWH would be expected. However, Semmens et al. (2015) did evaluate 4-h evening intervals (6 p.m.–10 p.m., 10 p.m. to 2 a.m.) pooling data from all homes in the study, from rural areas in Montana, Alaska, and the Navajo Nation. Their highest 4-h ambient PM_2.5_ concentrations were reported during the 10 p.m. to 2 a.m. interval (23 μg/m^3^ median) with 6 p.m.–10 p.m. next (21 μg/m^3^ median) (Semmens et al., 2015). In context to all these studies, Keene, NH appears to experience similar to somewhat lower winter seasonal averages (Keene average 24-h AQS intervals reported at 5.3–9.0 μg/m^3^ over the 4 seasons, Fig. S1), 1-h averages (PA units reporting from 4.79 to 11.56 μg/m^3^ over the 4 seasons, Table S5), and 8-h averages in the evening (PA units reporting from 4.93 to 15.66 μg/m^3^ over the 4 seasons, Table S6).

The many different time intervals selected across different research groups challenge any direct community to community PM comparisons. A major contribution of this study is the long-term measurement of exposure over 4 winters in a rural valley in northern New England and the detailed temporal analysis of exposure (hourly, daytime, evening, early morning, Figs. S8 and S9). There are few long term outdoor PM_2.5_ exposure assessments for rural communities impacted by woodsmoke in the Northeast. Fleisch et al. (2020) report on indoor PM_2.5_ for a cohort of 152 pregnant women in northern New England, finding a median PM_2.5_ concentration of 6.7 μg/m^3^ (7-day sampling interval) in those homes that used woodstoves. We recommend additional research of both indoor and outdoor PM_2.5_ in rural communities in the Northeast to better understand the exposure impact of woodsmoke on community health. Penn et al. (2017) attributed 10,000 additional U.S. deaths per year to RWH, identifying high impact from RWH in Northeast states due to population density and climate.

**Fig. 5.**
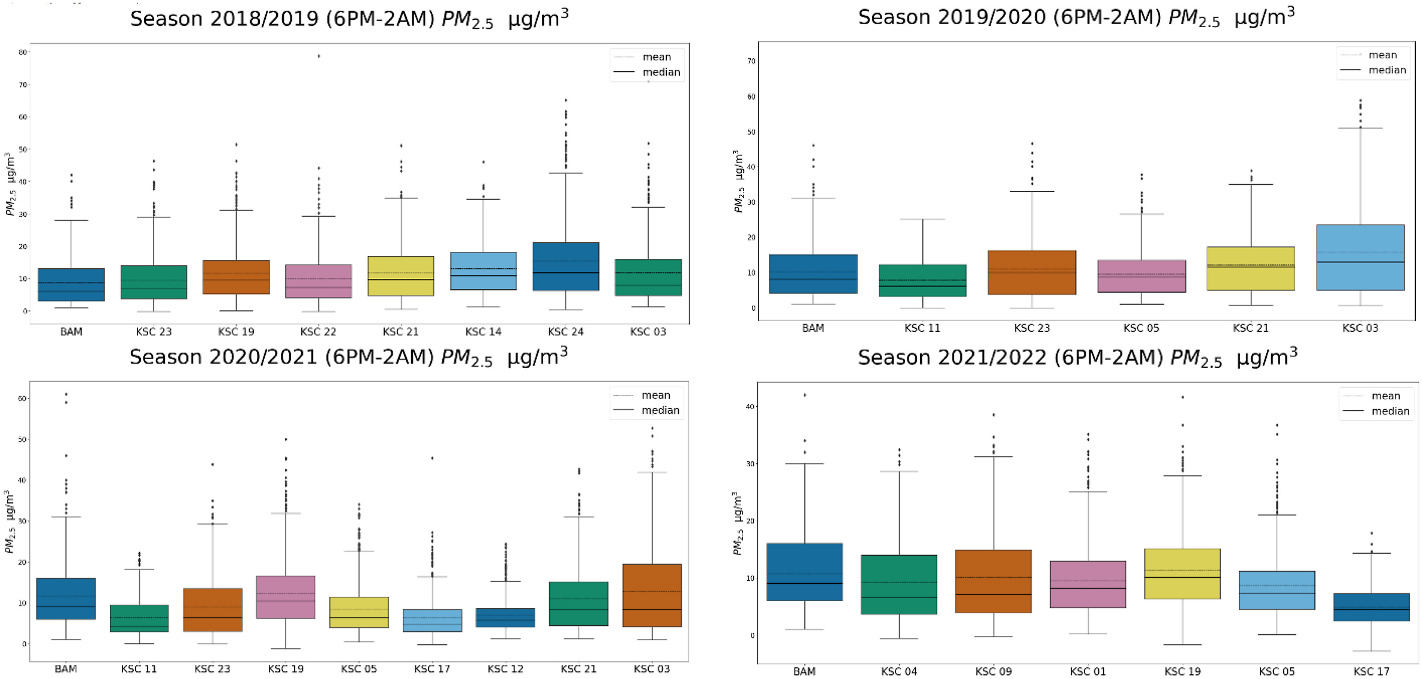
a, b, c, d from top left: Boxplots of 8-h intervals comparing PA PM_2.5_ per heating season in μg/m^3^ across the community network in Keene, NH from 2018 to 2022 plotted in order left to right of increasing distance away from the BAM (0–5000 m). The FEM BAM PM_2.5_ concentration is included for comparison, with KSC-23 being the collocated unit for season 2018/2019, KSC-23 and KSC-11 being the collocated units for season 2019/2020 and season 2020/2021, and KSC-09 and KSC-04 the collocated units for season 2021/2022. All other units were deployed in various regions throughout Keene, NH (Table S2).

This study supports that the selection of the averaging interval has large implications for better understanding exposure from RWH. We observed wintertime diurnal patterns of exposure across heating seasons consistently indicating that hourly PM averages peaked in the evening hours, whereas daytime hourly PM levels were minimal (<5 μg/m^3^). Elevated hourly and 8-h exposures occurred across the community throughout the 4-year study period (Figs. 4 and 5), with a maximum hourly interval of 81.7 μg/m^3^ (West Keene, KSC-24) and a maximum 8-h average up to 78.7 μg/m^3^ (North/Central Keene, KSC-22) in residential neighborhoods 800 m–5000 m away from the BAM. The current method for measuring compliance with the National Ambient Air Quality Standard for PM_2.5_ is a midnight to midnight 24-h average, a time interval which may not best reflect the variation in exposure observed in this study or in other communities impacted by RWH. Our results demonstrated that the BAM did not capture the spatial variability of woodsmoke exposure in the Keene community, with significantly higher PM_2.5_ in West Keene for 3 heating seasons for all time intervals (hourly, and 8-h daytime, evening, and late evening/early morning intervals, *p* < 0.001). Overall, the PA community-wide network effectively characterized long-term elevated PM exposure at the neighborhood scale, as well as identified short-term elevated exposures.

#### 3.3.4. Comparison to other Purple Air research studies

Most studies of PA sensors have mainly focused on the evaluation of sensor performance (Ardon-Dryer et al., 2020; Barkjohn et al., 2021; Malings et al., 2020; Wallace et al., 2021), with some studies reporting concentrations during high PM events like wildfires (Holder et al., 2020) or wintertime inversions in Salt Lake City, Utah (Ardon-Dryer et al., 2020). Fewer studies have reported long-term year over year concentrations in U.S. communities or the utility of a PA network for long-term exposure assessment. Connolly et al. (2022) deployed PAs in a Southern California community at UCLA over a 1.5-year period and concluded that PA sensors were a cost-effective way to monitor indoor and outdoor air quality with an engaged community, providing accurate, highly time resolved PM concentrations for multiple stakeholders. While their research group had lower data completeness (only 54 % for outdoor units), and worked within an urban airshed, our study supports their overall conclusions. We suggest that the higher data completeness the Keene study was able to achieve was due to having automated QA/QC checks every 24 h, with a team of undergraduate students and engaged citizens, many of which reported how they enjoyed working with the students. Having a group of students “on standby” enhanced timely troubleshooting and resolution of issues, leading to higher data completeness. While undergraduate student support was an overall project strength, there were still setbacks. A key West Keene sensor (KSC-03) was excluded for a heating season (less than 80 % data completeness) due to internet connection issues, like those reported in Connolly et al. (2022). Without a stable internet connection for PA sensors, data completeness goals may be challenging in future studies, particularly in disadvantaged communities. Additionally, participants sometimes inadvertently shut off power, or opted out of participation due to various difficult-to-predict concerns, such as concerns on PA electricity consumption. Keene’s longer history of multiple non-profits, schools, and state agencies doing public education about woodsmoke and proper wood burning techniques likely generated higher community engagement in hosting sensors.

Lu et al. (2022) also deployed a PA network of 22 sensors to characterize hourly PM exposure in eight communities in Southern California. Their study used PAs to identify “hotspots” in disadvantaged communities related to traffic sources and investigate community-level average exposure. Their research also determined temporal patterns of higher PM_2.5_ during the evenings and during the winter season (25.8 μg/m^3^, December 2021) possibly due to more wildfire activity or temperature inversions (Lu et al., 2022). Their study appears to document a timeline of “hot-spot” discovery and temporal pattern identification similar to the Keene Clean Air project milestones, supporting the benefits of low cost sensors to improve geospatial and temporal coverage in EJ communities. Over the years, the Keene Clean Air project implemented educational interventions in the community based on “hot-spot” identification and maintained an active local map since the project’s start, pre-dating the Purple Air website map. However, the current Purple Air website map, with drop-down menu functionality to apply various correction equations, and the EPA’s Air Now map (which includes public PAs), have made the Keene Clean Air map rather obsolete.

## 4. Conclusion

While the setting was in a small city in rural New Hampshire impacted by RWH, our study also supports the effectiveness and utility of implementing a low-cost Purple Air sensor network for long-term PM_2.5_ exposure assessment in other community settings. We provided practical “lessons learned” on QA/QC, sensor management, and correction model selection while deploying our network with citizen scientists in Keene, New Hampshire over 4 heating seasons. We found that local data driven models (MLR, quadratic fit, and Wallace (2023)) reduced overall bias in the complete dataset, but with unacceptable bias in concentrations over 35 μg/m^3^. This is likely due to the small number (n = 13) of actual BAM reference monitor readings >35 μg/m^3^ in this study. Therefore, we applied the EPA correction equation from Barkjohn et al. (2021), which had the lowest bias of all correction models for concentrations >35 μg/m^3^. We used GIS to develop a semi-quantitative map of concentrations which visually highlighted the spatial variability across the community at the neighborhood scale. This mapping approach quickly identified neighborhoods with higher PM_2.5_ concentrations, demonstrating the utility of PAs and GIS for “hot spot characterization” and producing useful public health data for local decision-making.

Using ANOVA, we determined significant differences in PM_2.5_ between the reference monitor and various neighborhoods across Keene, with many neighborhoods experiencing significantly increased PM_2.5_ exposure over multiple seasons, at both hourly and 8-h evening time intervals (*p* < 0.01). In the Keene case, the reference monitor didn’t capture spatial heterogeneity of exposure from RWH, likely due to the nature of woodsmoke exposure being driven by local sources at the neighborhood scale. Temperature inversions in the rural valley also increased PM concentrations during cold evenings with little to no wind. We suggest spatially heterogenous woodsmoke exposure at a neighborhood scale may occur in other rural valley communities in the Northeast, so we recommend additional research in RWH impacted communities. Corrected data from PA units accurately assessed PM exposure (2.4 % *NMBE*, 3.3 μg/m^3^ *RMSE*) in Keene and could prove useful for long-term exposure assessment for other communities impacted by RWH but with no FEM reference monitors nearby.

Consistent with other performance evaluations, we determined PA low-cost sensors have excellent precision, low bias post-correction (<15 %), report highly time resolved data, improve spatial coverage where FEM coverage is unavailable, and are stable over long time periods. Like conclusions by Lu et al. (2022) and Kramer et al. (2023), we believe our study supports the use of PA sensor networks to fill in spatial gaps of PM monitoring to provide timely data to disadvantaged communities and improve understanding of their long-term exposure. We recommend automating frequent ‘real time’ QA/QC checks of low-cost sensors. Future research is recommended to improve and apply spatial exposure modeling techniques to more accurately assess community-wide exposure with a limited number of low-cost sensors.

In consideration of their low-cost and durability even in New Hampshire winters, PA sensors can increase options for researchers and citizen scientists to assess long-term community exposure to air pollution and develop actionable data for policy solutions to improve community and regional health. These outcomes are best ensured within a community with engaged citizens, accessible expertise, and personnel resources to manage sensor troubleshooting and large database management. Finally, while this study used Purple Air sensors, there are other low-cost sensors and data management systems available for use in communities. Our paper is not a product endorsement for Purple Air.

## Supplementary Material

Traviss et al. 2025 AE Supp Info

Appendix A. Supplementary data

Supplementary data to this article can be found online at https://doi.org/10.1016/j.atmosenv.2025.121398.

## Figures and Tables

**Table 1:** Normalized Mean Bias Error (*NMBE*) for the models evaluated in this study. Red color represents underestimation of model compared to the FEM/BAM (reference monitor), and blue represents overestimation. The Wallace* model comparison used a local correction factor, as explained in Appendix B, Supplemental Information.

Correction Model	All PM data (n = 6454)	Good (0.0–9.0 μg/m^3^) (n = 4446)	Moderate (9.1–35.4 μg/m^3^) (n = 1995)	Unhealthy (35.5–125.4 μg/m^3^) (n = 13)
No Correction	**54.6%**	**36.8%**	**65.3%**	**78.2%**
EPA	**2.4%**	**13.0%**	**−4.0%**	**−3.4%**
Robinson	**−8.4%**	**−12.8%**	**−5.8%**	**−0.7%**
LRAPA	**−30.8%**	**−46.4%**	**−21.5%**	**−12.5%**
Nilson	**23.9%**	**10.4%**	**31.9%**	**42.3%**
AQ&U	**12.9%**	**30.6%**	**2.2%**	**−1.2%**
Wallace*	**0.0%**	**−3.9%**	**2.5%**	**−8.2%**
Local Quadratic	**0.0%**	**12.4%**	**−7.5%**	**−6.6%**
Local Linear	**0.0%**	**13.2%**	**−7.9%**	**−12.1%**

## Data Availability

Data will be made available on request.

## HIGHLIGHTS

- Long-term PM_2.5_ exposure data in woodsmoke-impacted U.S. rural valleys are limited.
- Low-cost sensors are a promising tool for exposure assessment in communities, but real-world data on implementation is needed.
- We determined significant PM_2.5_ differences over multiple winters between Purple Airs in neighborhoods and the Federal Equivalent Monitor.
- Purple Airs provided reliable data for 3 to 4 years in winter conditions using appropriate QA/QC and post-correction models.
