# Supplementary material for: Four-year community-wide PM2.5 exposure characterization using a low-cost sensor network in a rural valley influenced by residential wood smoke
Source: Atmos Environ (1994). Author manuscript; Available in PMC 2025 Nov 26. (PMC12646610; doi:10.1016/j.atmosenv.2025.121398)
Supplement: Traviss et al. 2025 AE Supp Info [file NIHMS2111320-supplement-Traviss_et_al__2025_AE_Supp_Info.docx]

**Supporting Information**

Title: **Four-year community-wide PM_2.5_ exposure characterization using a low-cost sensor network in a rural valley influenced by residential wood smoke**

Authors: Nora Traviss*^1,2^, John Stanway^1,2^, John Woodward^2^, Thomas Webler^2,4^, George Allen^1^, Mahdi Ahmadi^1,3^

[*ntraviss@nescaum.org](mailto:*ntraviss@nescaum.org), corresponding author

^1^ Northeast States for Coordinated Air Use Management (NESCAUM), Boston, MA, 02111, USA

^2^ Keene State College, Keene, NH, 03435, USA

^3^ University of North Texas, Denton, TX, 76203, USA

^4^ Social and Environmental Research Institute, Greenfield, MA 01301


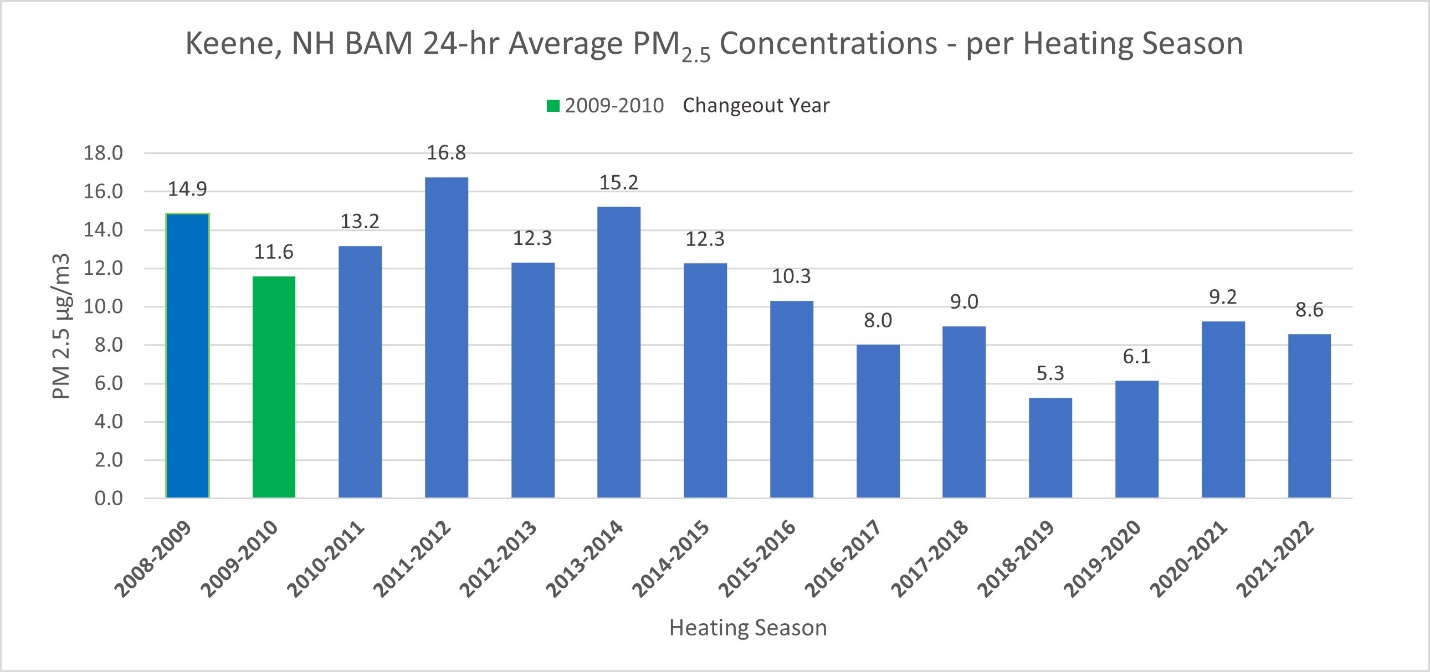


Figure S1: Daily mean PM_2.5_ (24-hour average) of Keene, NH Beta Attenuation Monitor (BAM) Federal Equivalent Method (FEM) for each heating season from 2008/2009 to 2022/2023. Heating season is defined as starting December 1 to February 28. A woodstove changeout of 86 stoves occurred in 2009/2010. Data downloaded from EPA Air Quality System, November 2023.


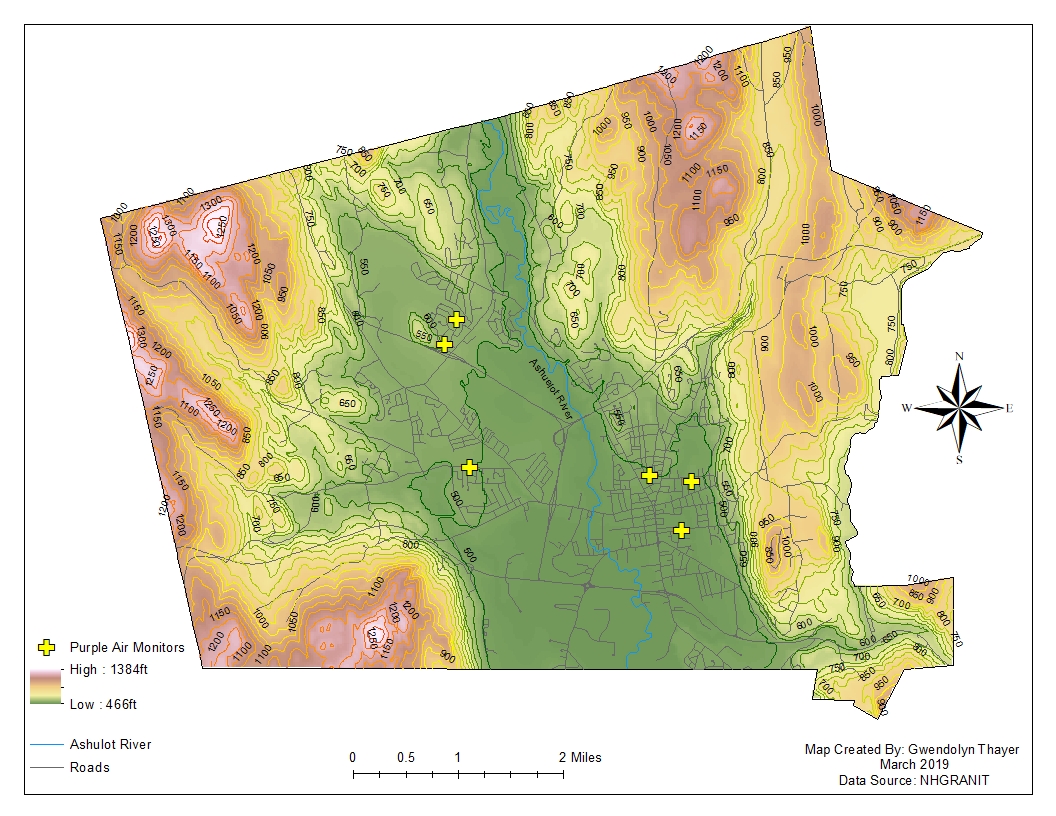
Figure S2: Topographical map (50 feet contour lines) of Keene, NH’s valley geography. The yellow cross represents Purple Air locations in the valley floor and the red “X” is a Purple Air at approximately 780 feet elevation. Map created by Gwendolyn Thayer Kimberling, as part of a Senior Capstone project at Keene State College. Source: Kimberling, T. G., and West, K. 2019. *Predicting Air Inversions Using Neighborhood Based Air Monitoring and Drone Technology: Part of a Program to Encourage Voluntary Reduction in Residential Wood Burning to Improve Air Quality in Keene, NH. Environmental Studies Department. Unpublished. Available from the corresponding author upon request.*


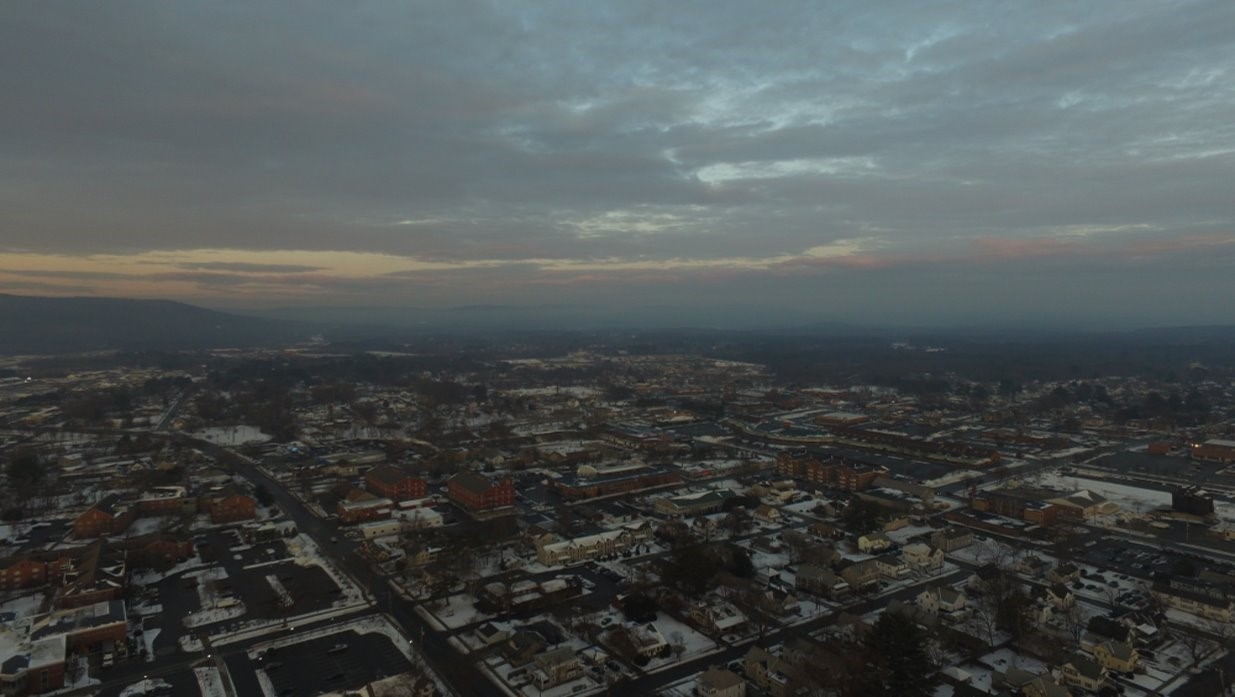


| Figures S3: Photo of air pollution during temperature inversion occurring February 4, 2019, taken 7:06 am, height 400 ft, via drone flown from Fiske Quad at Keene State College. Direction is ~ northwest of college campus. (Source: John Woodward). |
| --- |


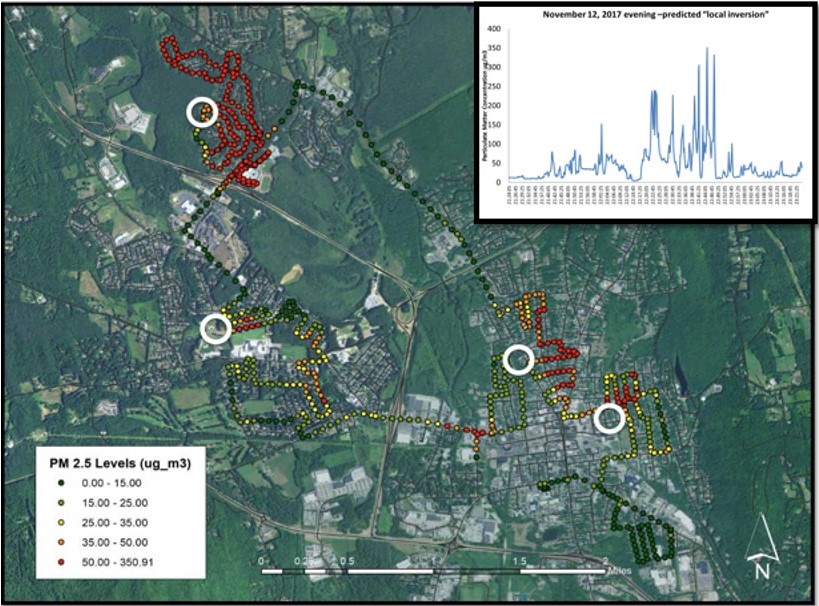


Figure S4: Mobile monitoring map of the evening of November 12, 2017 (inset), with 10-second measurements of PM_2.5_ taken using a Thermo-PDR1500. Data collected and map prepared and documented as part of senior capstone project at Keene State College. Source: Clark, A., Jones B., Kraft A., and Linera B. (2018). *Beyond the Haze: An Assessment of Air Quality in Keene, New Hampshire* Keene State College. Environmental Studies Department. *Unpublished. Available from the corresponding author upon request.*

## Hourly (1-hour interval) PM_2.5_ Time Series

| 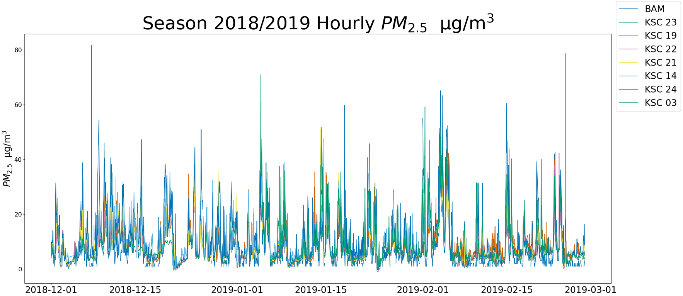 | 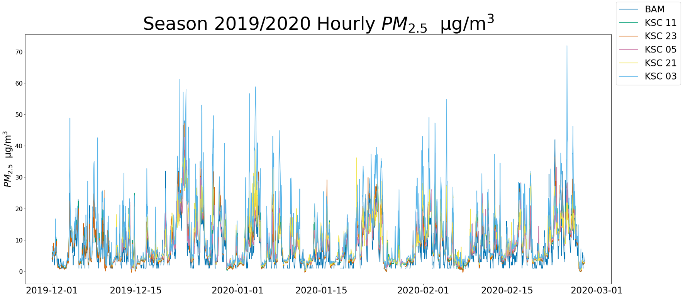 |
| --- | --- |
| 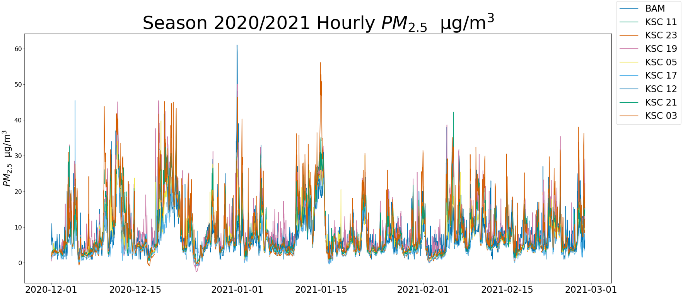 | 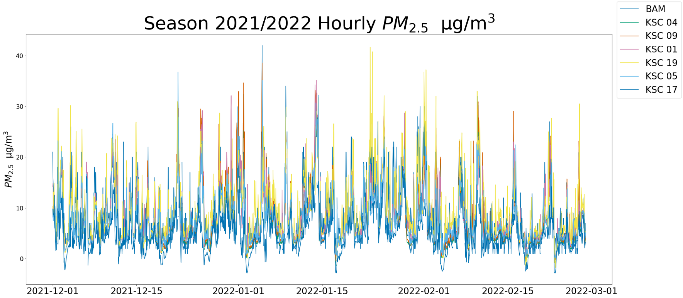 |
| Figure S5 a, b, c, d, from top left: Time series analysis of PM_2.5_ measurements (in µg/m^3^) in the heating seasons in Keene, NH from the year 2018 to 2022. The sustained peaks in the time series likely represent temperature inversion episodes that occurred within the valley in Keene. See Table S2 for a key to sensor location. | |

## Hourly (1-hour) PM_2.5_ Boxplots – Each Heating Season

| 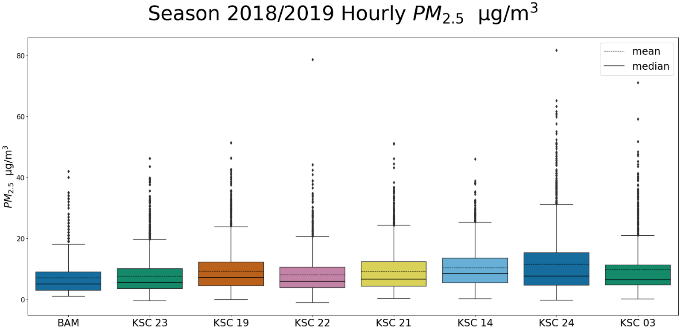 | 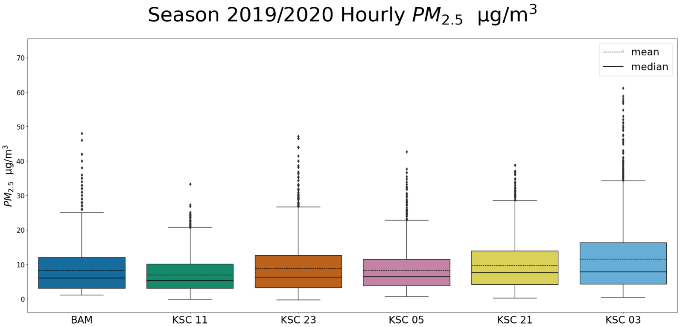 |
| --- | --- |
| 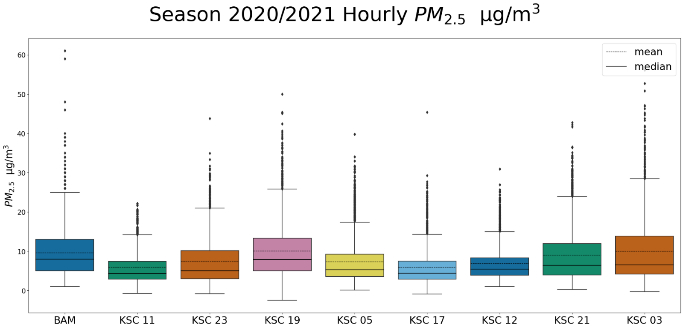 | 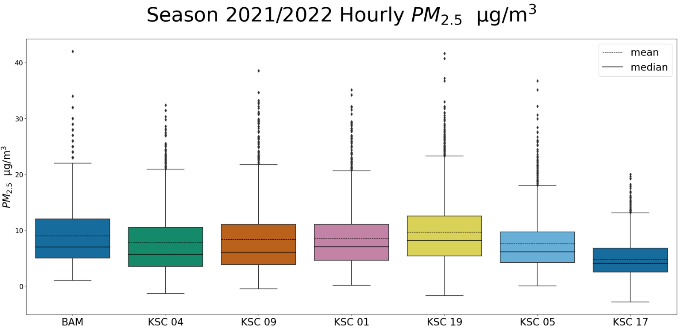 |
| Figure S6 a, b, c, d, from top left: Boxplots of hourly PM_2.5_ (in µg/m^3^) in the heating seasons in Keene, NH from the years 2018 to 2022, plotted by increasing distance away from the BAM. The mean is the dotted line and the median is the solid line. The FEM BAM PM_2.5_ concentration is also included for comparison, with KSC-23 being the collocated unit for season 2018/2019, KSC-23 and KSC-11 being the collocated units for season 2019/2020 and season 2020/2021, and the collocated units for season 2021/2022 were KSC-09 and KSC-04. See Table S2 for a key to sensor location. | |

## Evening 8-hour (6 pm to 2 am interval) PM_2.5_ Boxplots

| 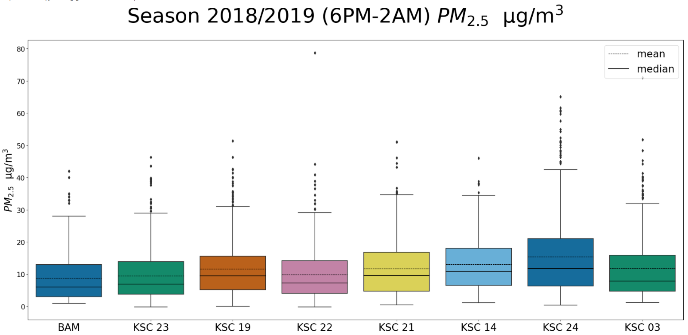 | 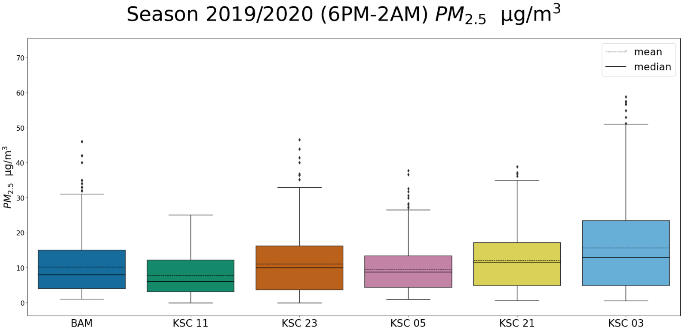 |
| --- | --- |
| 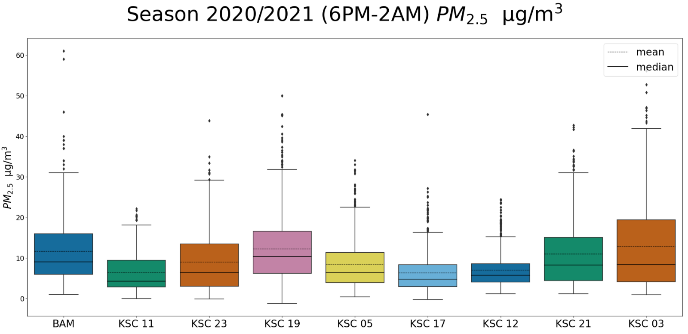 | 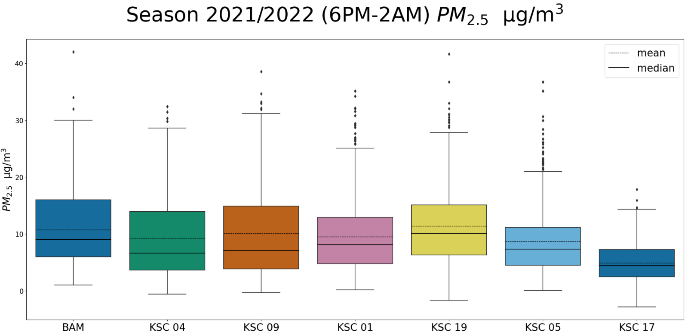 |
| Figure S7 a, b, c, d, from top left: Boxplots of 8-hour evening PM_2.5_ concentrations in µg/m^3^ across the community network in Keene, NH for four heating seasons. The mean is the dotted line and the median is the solid line. The FEM BAM PM_2.5_ concentration is also included for comparison, with KSC-23 being the collocated unit for season 2018/2019, KSC-23 and KSC-11 being the collocated units for season 2019/2020 and season 2020/2021, while the collocated units for season 2021/2022 were KSC-09 and KSC-04. All other units were deployed in various regions throughout Keene, NH, in order of distance away from the BAM. See Table S2 for a key to sensor location. | |

## Daytime 8-hour (10 am to 6 pm interval) PM_2.5_ Boxplots

| 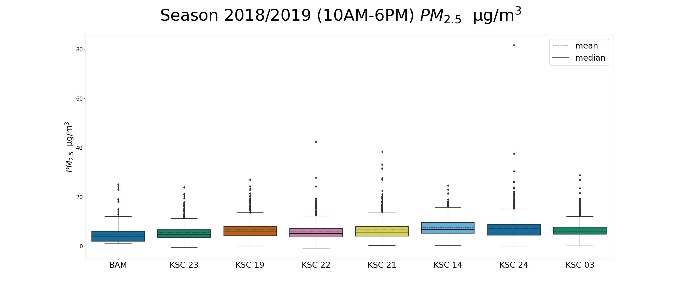 | 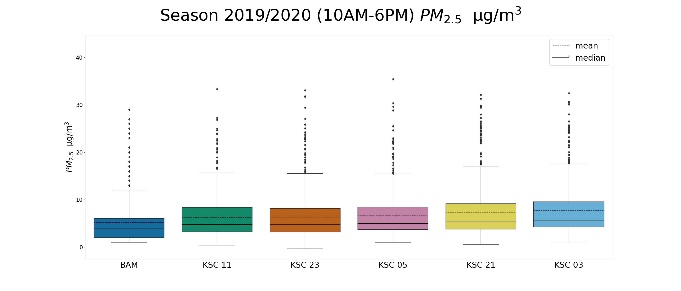 |
| --- | --- |
| 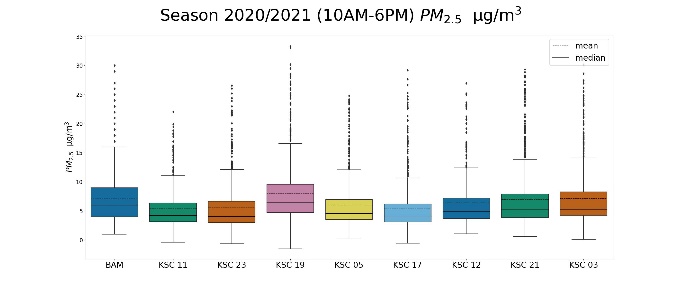 | 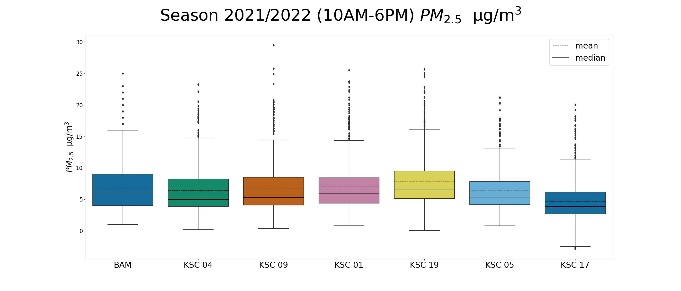 |
| Figure S8 a, b, c, d, from top left: Boxplots of 8-hour daytime PM_2.5_ concentrations in µg/m^3^ across the community network in Keene, NH for four heating seasons. The mean is the dotted line and the median is the solid line. The FEM BAM PM_2.5_ concentration is also included for comparison, with KSC-23 being the collocated unit for season 2018/2019, KSC-23 and KSC-11 being the collocated unit for season 2019/2020 and season 2020/2021, while the collocated units for season 2021/2022 were KSC-09 and KSC-04. All other units were deployed in various regions throughout Keene, NH in order of distance away from the BAM. See Table S2 for a key to sensor location. | |

## Late Evening/Early Morning 8-hour (2 am to 10 am interval) PM_2.5_ Boxplots

| 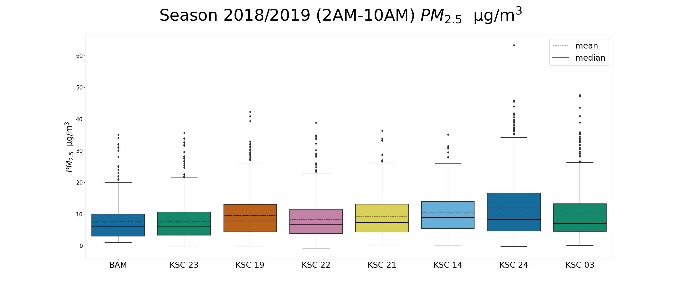 | 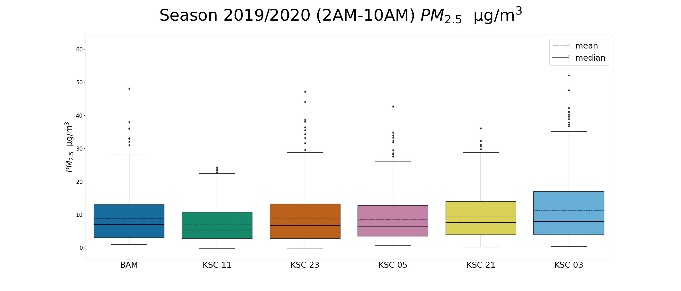 |
| --- | --- |
| 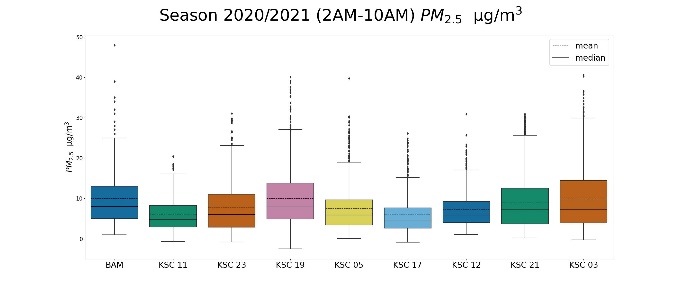 | 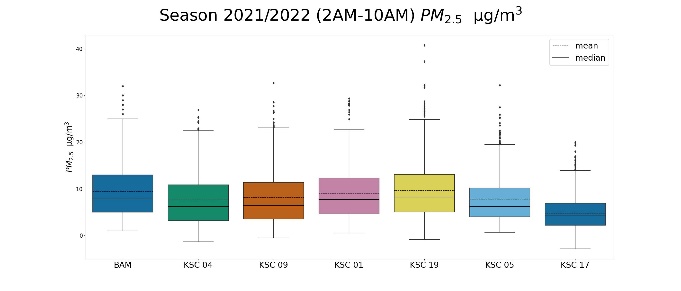 |
| Figure S9 a,b,c,d, from top left: Boxplots of 8-hour late evening/early morning PM_2.5_ concentrations in µg/m^3^ across the community network in Keene, NH for four heating seasons. The mean is the dotted line and the median is the solid line. The FEM BAM PM_2.5_ concentration is also included for comparison, with KSC-23 being the collocated unit for season 2018/2019, KSC-23 and KSC-11 being the collocated unit for season 2019/2020 and season 2020/2021, while the collocated units for season 2021/2022 were KSC-09 and KSC-04. All other units were deployed in various regions throughout Keene, NH in order of distance away from the BAM. See Table S2 for a key to sensor location. | |

## Table S1: Comparison of Correction Models, Normalized RMSE by Model and Concentration Range.


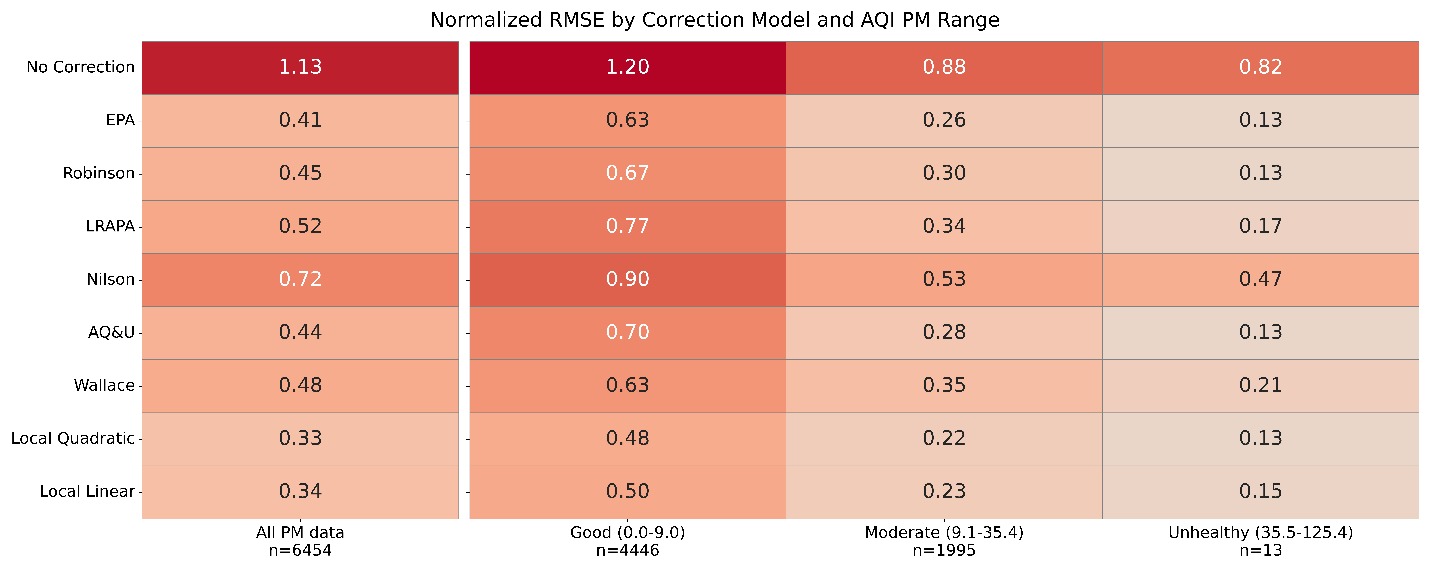


## Table S2: Purple Air Sensor Location and Distance from the BAM, per Heating Season

| Heating Season 2018 - 2019 | | |  | Heating Season 2019 - 2020 | | |
| --- | --- | --- | --- | --- | --- | --- |
| Sensor Unit | Neighborhood | Distance from BAM |  | Sensor Unit | Neighborhood | Distance from BAM |
| BAM | Water Street | 0 meters |  | BAM | Water Street | 0 meters |
| KSC-23 | Water Street, FEM co-location | 0 meters |  | KSC-23 | Water Street, FEM co-location | 0 meters |
| KSC-19 | East Keene, Beech Street | 800 meters |  | KSC-21 | West Keene, Keene High School | 3500 meters |
| KSC-21  KSC-14 | West Keene, Keene High School  West Keene, Near High School | 3500 meters  4000 meters |  | KSC-03 | West Keene, Maple Acres | 5000 meters |
| KSC-22 | North/central, Union Street | 1000 meters |  | KSC-05 | East Keene, Martin Street | 900 meters |
| KSC-24 | West Keene, Maple Acres | 5000 meters |  | KSC-11 | Water Street, FEM co-location | 0 meters |
|  |  |  |  |  |  |  |
| Table S2 (continued) | | |  | Table S2 (continued) | | |
| Heating Season 2020 - 2021 | | |  | Heating Season 2021 - 2022 | | |
| Sensor Unit | Neighborhood | Distance from BAM |  | Sensor Unit | Neighborhood | Distance from BAM |
| BAM | Water Street | 0 meters |  | BAM | Water Street | 0 meters |
| KSC--23 | Water Street, FEM co-location | 0 meters |  | KSC-19 | East Keene, Beech Street | 800 meters |
| KSC-19 | East Keene, Beech Street | 800 meters |  | KSC-05 | East Keene, Martin Street | 900 meters |
| KSC-21 | West Keene, Keene High School | 3500 meters |  | KSC-17 | East Keene, Roxbury Street (elevated: 780 feet) | 1400 meters |
| KSC-03 | West Keene, Maple Acres | 5000 meters |  | KSC-01 | Central Keene, Monadnock Street | 650 meters |
| KSC-05 | East Keene, Martin Street | 900 meters |  | KSC-04 | Water Street, FEM co-location | 0 meters |
| KSC-11 | Water Street, FEM co-location | 0 meters |  | KSC-09 | Water Street, FEM co-location | 0 meters |
| KSC-12 | North Keene, Woodbury Street | 2000 meters |  |  |  |  |
| KSC-17 | East Keene, Roxbury Street (elevated: 780 feet) | 1400 meters |  |  |  |  |

## Table S3: PA Sensor Limit of Detection (LOD)

| **Sensor** | **LOD** |
| --- | --- |
| KSC-23 | 1.73 |
| KSC-19 | 0.19 |
| KSC-22 | 0.60 |
| KSC-21 | 6.11 |
| KSC-24 | 0.23 |
| KSC-03 | 0.08 |
| KSC-13 | 0.09 |
| KSC-14 | 0.21 |
| KSC-11 | 2.00 |
| KSC-05 | 0.06 |
| KSC-17 | 2.21 |
| KSC-12 | 0.11 |
| KSC-04 | 3.23 |
| KSC-09 | 0.19 |
| KSC-01 | 0.53 |

## Table S4: References for other Correction Methods

| **Correction Method** | **Equation** |
| --- | --- |
| 1. Barkjohn et al., 2021 | $PM_{Corrected}=0.524 *PA_{cf1}-0.0862*PA_{rh}+5.75$ |
| 1. Robinson et al., 2020 | $PM_{Corrected}=0.55*PA_{cf1}$ |
| 1. Matonte et al., 2020 | $PM_{Corrected}=0.5*PA_{cf1}-0.66$ |
| 1. Nilson et. al., 2022 | $PM_{Corrected}=\frac{\left( PM_{cf1} \right)}{1+\frac{0.24}{\frac{100}{PA_{rh}}-1}}$ |
| 1. Ardon-Dryer et al., 2020 | $PM_{Corrected}=0.778*PA_{cf1}+2.65$ |

## Table S5: Descriptive Statistics Tables for Hourly PM_2.5_ µg/m^3^

| **Hourly** | | | | | | | | | | |
| --- | --- | --- | --- | --- | --- | --- | --- | --- | --- | --- |
| **Season 2018-2019** | | | | |  | **Season 2019-2020** | | | | |
| unit | Std error of mean | mean | median | max |  | unit | Std error of mean | mean | median | max |
| KSC-03 | 0.22 | 9.73 | 6.43 | 71.04 |  | KSC-03 | 0.22 | 11.56 | 7.83 | 71.92 |
| KSC-14 | 0.18 | 10.38 | 8.39 | 46.04 |  | KSC-05 | 0.14 | 8.28 | 6.40 | 42.71 |
| KSC-19 | 0.17 | 9.32 | 7.08 | 51.37 |  | KSC-11 | 0.12 | 7.00 | 5.21 | 33.34 |
| KSC-21 | 0.16 | 9.17 | 6.64 | 51.11 |  | KSC-21 | 0.16 | 9.66 | 7.61 | 38.84 |
| KSC-22 | 0.16 | 8.11 | 5.93 | 78.70 |  | KSC-23 | 0.17 | 8.76 | 6.25 | 47.14 |
| KSC-23 | 0.14 | 7.59 | 5.54 | 46.28 |  | BAM | 0.17 | 8.29 | 6.00 | 48.00 |
| KSC-24 | 0.22 | 11.50 | 7.55 | 81.69 |  |  |  |  |  |  |
| BAM | 0.15 | 7.11 | 5.00 | 42.00 |  |  |  |  |  |  |
| **Season 2020-2021** | | | | |  | **Season 2021-2022** | | | | |
| unit | Std error of mean | mean | median | max |  | unit | Std error of mean | mean | median | max |
| KSC-03 | 0.18 | 10.04 | 6.51 | 56.09 |  | KSC-01 | 0.12 | 8.54 | 7.04 | 35.14 |
| KSC-05 | 0.12 | 7.35 | 5.34 | 39.78 |  | KSC-04 | 0.16 | 7.79 | 5.69 | 32.40 |
| KSC-11 | 0.13 | 5.92 | 4.31 | 22.15 |  | KSC-05 | 0.10 | 7.63 | 6.13 | 36.73 |
| KSC-12 | 0.12 | 6.92 | 5.48 | 30.92 |  | KSC-09 | 0.16 | 8.36 | 6.05 | 38.57 |
| KSC-17 | 0.10 | 5.93 | 4.41 | 45.41 |  | KSC-17 | 0.08 | 4.79 | 4.08 | 20.04 |
| KSC-19 | 0.16 | 10.10 | 7.89 | 50.01 |  | KSC-19 | 0.13 | 9.65 | 8.16 | 41.64 |
| KSC-21 | 0.16 | 8.98 | 6.45 | 42.72 |  | BAM | 0.13 | 9.02 | 7.00 | 42.00 |
| KSC-23 | 0.15 | 7.40 | 4.99 | 43.82 |  |  |  |  |  |  |
| BAM | 0.15 | 9.65 | 8.00 | 61.00 |  |  |  |  |  |  |
| These are the descriptive statistics for each Purple Air unit used within each heating season. The tables include the mean, median, max recorded PM_2.5_ level and standard error of mean. This table uses all hourly readings that passed the QA/QC steps. | | | | | | | | | | |

### Table S6: Descriptive Statistics for 8-hour Interval, 6pm to 2am PM_2.5_ µg/m^3^

| **6pm to 2am** | | | | | | | | | | |
| --- | --- | --- | --- | --- | --- | --- | --- | --- | --- | --- |
| **Season 2018-2019** | | | | |  | **Season 2019-2020** | | | | |
| sensor | Std error of mean | mean | median | max |  | sensor | Std error of mean | mean | median | max |
| KSC-03 | 0.45 | 11.82 | 7.89 | 71.04 |  | KSC-03 | 0.46 | 15.66 | 12.98 | 71.92 |
| KSC-14 | 0.38 | 13.11 | 10.95 | 46.04 |  | KSC-05 | 0.24 | 9.63 | 8.76 | 37.72 |
| KSC-19 | 0.36 | 11.68 | 9.55 | 51.37 |  | KSC-11 | 0.24 | 7.82 | 6.03 | 25.03 |
| KSC-21 | 0.34 | 11.75 | 9.59 | 51.11 |  | KSC-21 | 0.31 | 12.11 | 11.57 | 38.84 |
| KSC-22 | 0.33 | 9.87 | 7.20 | 78.70 |  | KSC-23 | 0.34 | 11.04 | 9.93 | 46.58 |
| KSC-23 | 0.30 | 9.49 | 6.95 | 46.28 |  | BAM | 0.31 | 10.22 | 8.00 | 46.00 |
| KSC-24 | 0.47 | 15.46 | 11.76 | 65.15 |  |  |  |  |  |  |
| BAM | 0.31 | 8.72 | 6.00 | 42.00 |  |  |  |  |  |  |
| **Season 2020-2021** | | | | |  | **Season 2021-2022** | | | | |
| sensor | Std error of mean | mean | median | max |  | sensor | Std error of mean | mean | median | max |
| KSC-03 | 0.40 | 12.84 | 8.35 | 56.09 |  | KSC-01 | 0.23 | 9.48 | 8.14 | 35.14 |
| KSC-05 | 0.23 | 8.48 | 6.38 | 34.01 |  | KSC-04 | 0.33 | 9.26 | 6.62 | 32.40 |
| KSC-11 | 0.26 | 6.37 | 4.25 | 22.15 |  | KSC-05 | 0.21 | 8.63 | 7.32 | 36.73 |
| KSC-12 | 0.19 | 7.04 | 5.71 | 24.43 |  | KSC-09 | 0.35 | 10.09 | 7.10 | 38.57 |
| KSC-17 | 0.19 | 6.35 | 4.81 | 45.41 |  | KSC-17 | 0.14 | 4.93 | 4.45 | 17.84 |
| KSC-19 | 0.31 | 12.29 | 10.39 | 50.01 |  | KSC-19 | 0.25 | 11.36 | 10.08 | 41.64 |
| KSC-21 | 0.32 | 11.01 | 8.23 | 42.72 |  | BAM | 0.26 | 10.72 | 9.00 | 42.00 |
| KSC-23 | 0.30 | 8.98 | 6.39 | 43.82 |  |  |  |  |  |  |
| BAM | 0.31 | 11.66 | 9.00 | 61.00 |  |  |  |  |  |  |
| These are the descriptive statistics for each Purple Air unit used within each heating season. The tables include the mean, median, max recorded PM_2.5_ level and standard error of mean. This table utilizes the 8-hour averages between the 6 pm and 2 am timeframe that passed the QA/QC steps. | | | | | | | | | | |


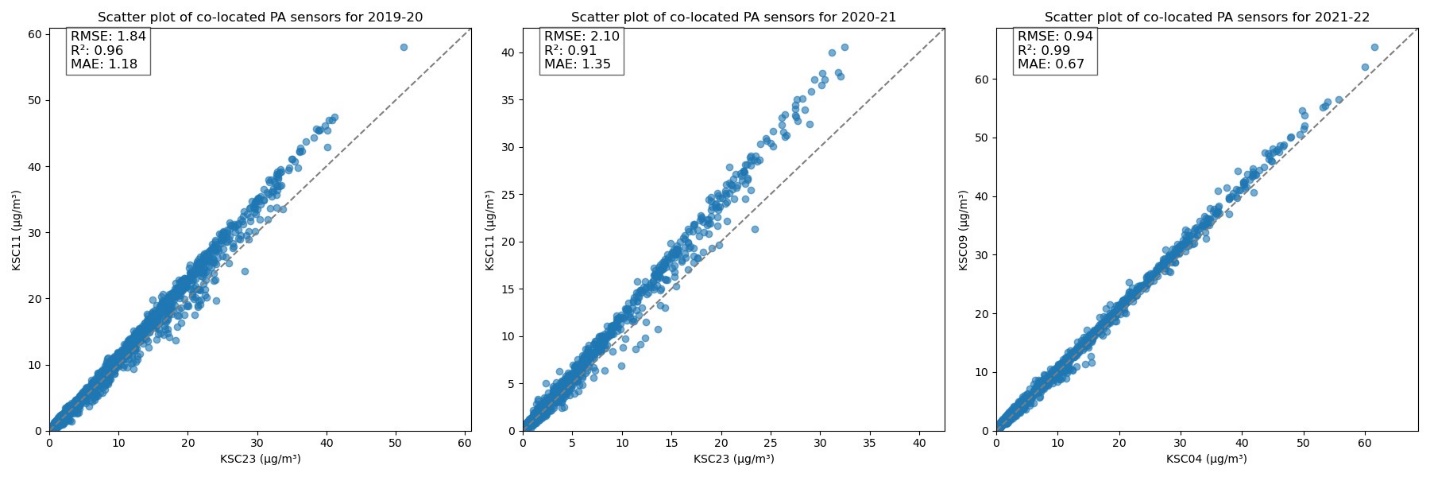


## Figure S10: Linear Regression of Collocated PA Units for the 2019/2020, 2020/2021, and 2021/2022 Seasons


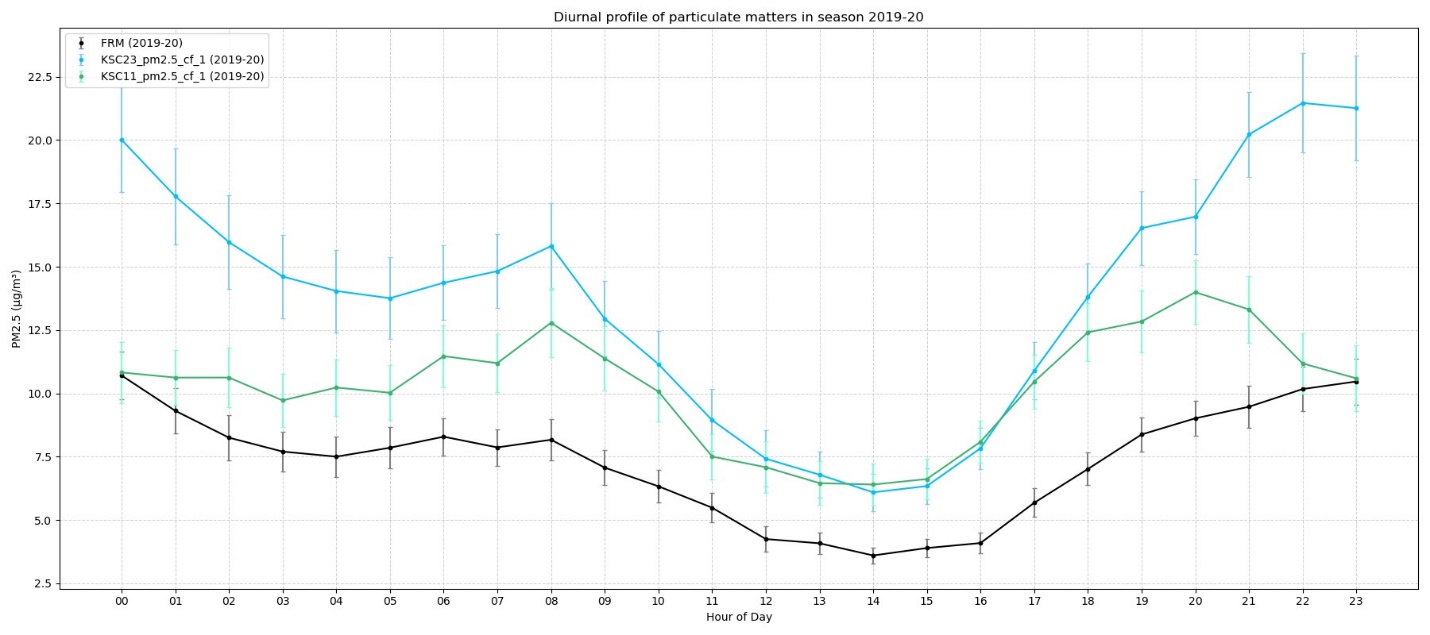


Figure S11 (a) KSC-23, KSC-11, and the BAM 1-hour averages for each hour per day, over the 2019/2020 heating season.


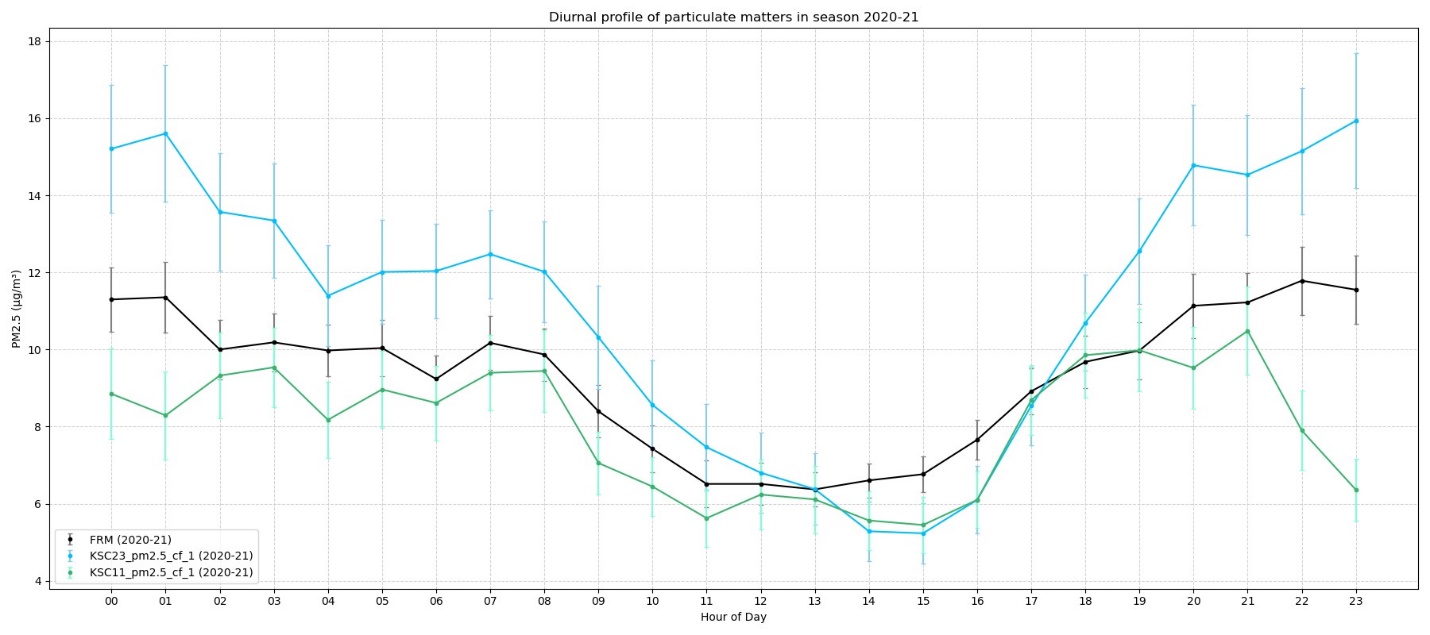


Figure S11 (b) KSC-23, KSC-11, and the BAM 1-hour averages for each hour per day, over the 2020/2021 heating season.
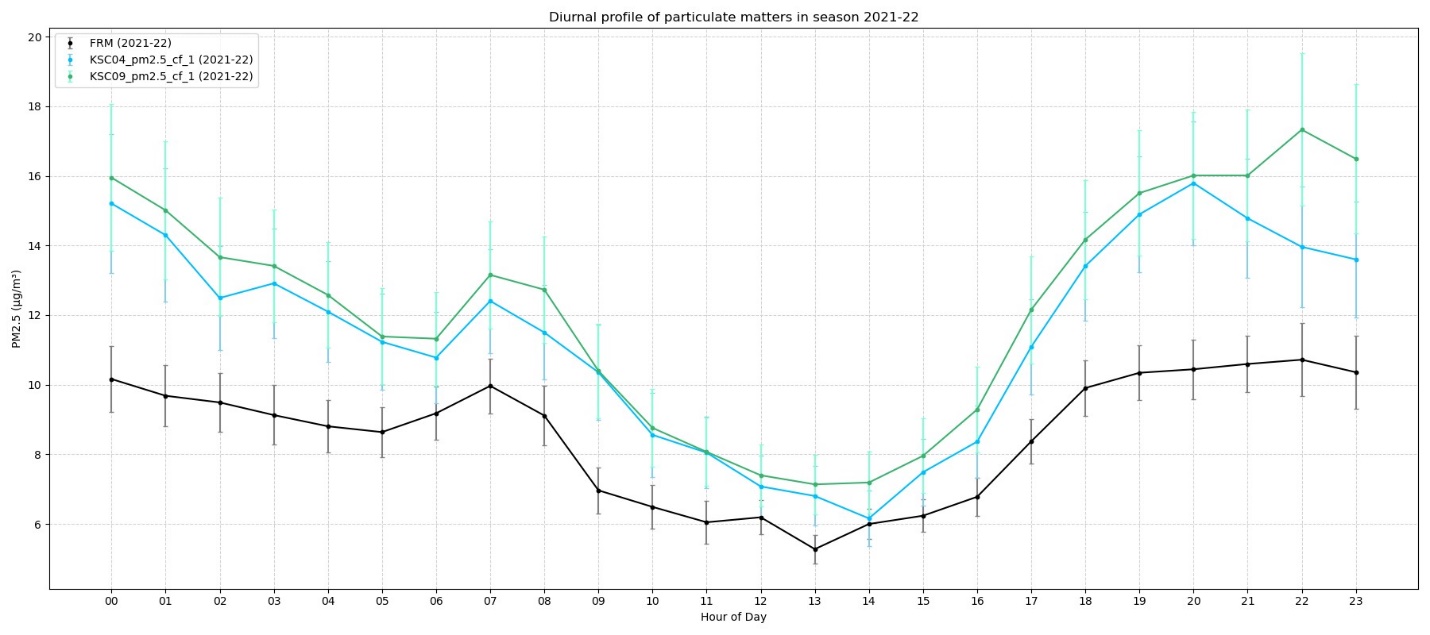


Figure S11 (c) KSC-04, KSC-09, and the BAM 1-hour averages for each hour per day, over the 2021/2022 heating season.

## Figure S11 a, b, c: Diurnal Profiles of PM_2.5_ Comparing BAM and Collocated PA Sensors for Seasons (a) 2019/2020; (b) 2020/2021; (c) 2021/2022.

Appendix A – Community Background and History of Citizen Science Research Collaborations, in Keene, NH

Keene, NH is a valley community long considered an area of concern for woodsmoke by the New Hampshire Department of Environmental Services (NHDES) due to elevated wintertime PM_2.5_ concentrations and 20% of residents reporting use of solid wood for heating (NHDES 2012, NHDES 2018, NHDES 2021). Keene also scores comparatively higher than other communities in New Hampshire on a state social vulnerability index due to various factors such as neighborhoods with high levels of poverty (96^th^ percentile in the U.S.), single parent households (90^th^ percentile), and disabled and elderly populations (90^th^ percentile) (NH Department of Health and Human Services, New Hampshire Social Vulnerability Index (SVI) Dashboard, 2024).

Elevated 24-hour PM_2.5_ levels exceeding 30 µg/m^3^ were noted after the NHDES implemented an hourly BAM Federal Equivalent Monitor alongside the traditional PM_2.5_ filter-based method in early 2009. A Woodstove Changeout Program during the 2009-to-2010-time frame replaced 86 stoves in the community (NHDES, 2010). After the changeout, PM_2.5_ concentrations in Keene reported by the BAM paradoxically increased in the subsequent heating seasons with the season average (based on 24-hour averages) reaching a maximum of 16.8 µg/m^3^ in the 2011/2012 heating season (Figure S1).

Since the 2009/2010 woodstove changeout, air quality monitoring activities and community outreach continued and involved multiple partners, such as NHDES, Keene State College, Southwest Region Planning Commission, and Cheshire Medical Center. Public outreach sessions on woodsmoke health hazards and proper wood storage and burning techniques were held once or twice every year from 2013 to 2020 at Keene State College and other venues. In 2018, the KSC team installed PA units at citizen scientist homes through a program called the Keene Clean Air project ([www.keenecleanair.org](http://www.keenecleanair.org)). Since that time, PA units sited across Keene transmitted data to the cloud and were subsequently input to an ARC-GIS mapping platform to report corrected PA concentration data at 10-minute intervals as a “snapshot” on a customized map on the publicly accessible website.

Over time, citizens from the outreach workshops were recruited as volunteers to work with Keene State College students and faculty to host PA monitors in different neighborhoods throughout the city. Through word-of-mouth, and the creation of the Keene Clean Air project website and Facebook page, new citizen scientists would ask to participate in the project and would be selected in different neighborhoods. We assessed neighborhoods for monitoring based on previous data gathered during the activities outlined in Figure S12, such as earlier mobile monitoring campaigns that identified PM hot spots (Figure S4), visual photo documentation of inversions (Figure S3), woodpile surveys, housing density, and student research projects that evaluated Keene’s topography (Figure S2). Keene State College students and faculty helped install outdoor PA units at citizen scientist homes in these neighborhoods. Research was conducted following Keene State College Institutional Review Board policies.


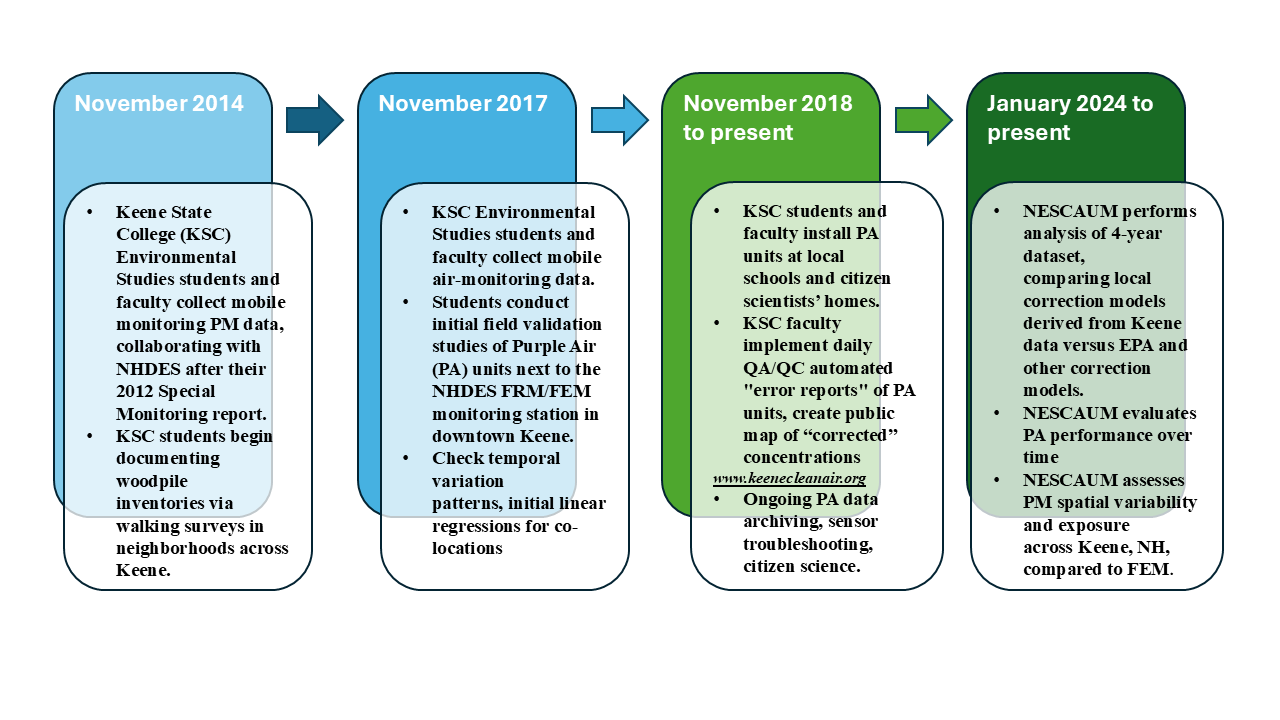


Figure S12 – History and timeline of Keene Clean Air associated research activities

Appendix B: Approach to Purple Air Correction Model Comparisons (referenced from Methods Section 2.2) and additional QA/QC results

As part of our work, we conducted a detailed evaluation of locally-derived correction models (based on PA data collocated next to the BAM reference monitor in Keene, NH) as well as evaluating correction models from the literature listed in Table S4. These models were based on PM_2.5_ concentration data. Additionally, we also derived a correction model based on particle number concentration as described by Wallace et al. (2021) and Wallace (2023).

As described previously in Methods Section 2.2, we used the PM_2.5_ CF_1 a and b channels at the 2-minute time interval based on previous research by Barkjohn et al. 2021 and Wallace et al. 2021 indicating lower bias for CF_1 versus CF_ATM. We used the particle number concentration data (> 0.3 µm, >0.5 µm, >1.0 µm, and >2.5 µm) in accordance with the steps outlined in Wallace et al. 2021 and Wallace 2023. For all CF_1 a and b concentration data, we adapted and applied the following cleaning procedures described in Table 1 in Connolly et al. (2022), which follows the steps outlined in Barkjohn et al. (2021), briefly summarized again here: we excluded data at the 2-minute interval if the a or b channel differed by more than 10 µg/m^3^, if the difference between the channels exceeded 5 µg/m^3^ and 61%, or if any negative PA values were reported. There were also temperature and humidity screens as outlined in Connolly et al. 2022. In our study, there were no values in the study that ever exceeded 100 µg/m^3^.

Next, the CF_1 a and b channel 2-minute data were averaged for each PA unit per heating season (defined as December 1 through February 28) and averaged again into hourly averages to match the BAM 1-hour intervals. Each 1-hour interval required a minimum of 24 2-minute readings (out of 30) to be included for further analysis, which ensured 80% completeness criteria. A Purple Air unit needed to meet 80% completeness to continue in the analysis. Collocated PA hourly averages were time-aligned with the hourly averages from the BAM. We removed data where the BAM concentration was below 0 µg/m^3^. The hourly average time-aligned data were used to both compare various correction methods (Table S4) and to develop two data-driven models, which we call “local linear” and “local quadratic.”

In total, we compared the performance of eight PA correction models using 1-hour averages. Five models were chosen from the literature (Table S4), we developed two models and applied the Wallace model (based on particle number concentration with local data). For the local models, we used the BAM PM, collocated PA PM and relative humidity (RH) data and fitted a linear and a quadratic model (Eq S1 and S2). We called these “local” models because the model coefficients were derived only from Keene collocated measurements. The local quadratic model includes relative humidity and a PM interaction term. Both local models were fitted separately for each heating season.

$$Local Linear model: PM_{2.5}=b_{0}+b_{1}\times PM_{PA}+b_{2}\times RH_{PA} (Eq S1)$$

$$Local Quadratic: PM_{2.5}=b_{0}+b_{1}\times PM_{PA}+b_{2}\times RH_{PA}+b_{3}\times PM_{PA}^{2}+b_{4}\times PM_{PA}\times RH_{PA} \left( Eq S2 \right)$$

Next, we evaluated model performance. Let *δ _y, rms_* (root-mean-square-deviation with units of y, µg/m^3^ and hereafter referred to as *RMSE*), normalized *RMSE (NRSME)*, mean bias error (*MBE*) and normalized *MBE (NMBE)* be defined in the following table, where P = predicted value and O = the observed value, shown in Table S7:

Table S7: Equations used in correction model evaluation

| $\boldsymbol{\delta y,rms=}\sqrt{\frac{\boldsymbol{1}}{\boldsymbol{n}}\sum_{\boldsymbol{i=1}}^{\boldsymbol{n}} \left( \boldsymbol{P}_{\boldsymbol{1}}\boldsymbol{-}\boldsymbol{O}_{\boldsymbol{i}} \right)^{\boldsymbol{2}}}$ | ***(Eq S3)*** |
| --- | --- |
| ***NRSME = 100% ****$\frac{\sqrt{\frac{\boldsymbol{1}}{\boldsymbol{n}}\sum_{\boldsymbol{i=1}}^{\boldsymbol{n}} \left( \boldsymbol{P}_{\boldsymbol{i}}\boldsymbol{-}\boldsymbol{O}_{\boldsymbol{i}} \right)^{\boldsymbol{2}}}}{\bar{\boldsymbol{O}}}$ | ***(Eq S4)*** |
| ***MBE =*** $\frac{\boldsymbol{1}}{\boldsymbol{n}}\sum_{\boldsymbol{i=1}}^{\boldsymbol{n}} \left( \boldsymbol{P}_{\boldsymbol{i}}\boldsymbol{-}\boldsymbol{O}_{\boldsymbol{i}} \right)$ | ***(Eq S5)*** |
| ***NMBE = 100% **** $\frac{\frac{\boldsymbol{1}}{\boldsymbol{n}}\sum_{\boldsymbol{i=1}}^{\boldsymbol{n}} \left( \boldsymbol{P}_{\boldsymbol{i}}\boldsymbol{-}\boldsymbol{O}_{\boldsymbol{i}} \right)}{\bar{\boldsymbol{O}}}$ | ***(Eq S6)*** |

The Leave One Out cross validation strategy was employed to calculate *r^2^, RMSE, NRMSE, MBE, and NMBE* for each of the collocated PA units per heating season over four seasons. Because there were two collocated units for all heating seasons (except 2018/2019), we selected the individual correction equation for the next three heating seasons based on the collocated unit with the lowest *MBE* and *RMSE* and highest *r^2^* per season.

To compare the performance of the local data-driven models to other correction models in Table S4, we applied the selected models to the raw PA CF_1 collocated data and determined their *r^2^, RMSE, NRMSE, and NMBE*. We also tested the correction method developed by Wallace et al. (2021), which uses particle number concentrations and not the CF_1 channel, by using the Keene PA particle number concentrations in equation (7) of Wallace (2023) for each collocated PA unit, across seasons. In our initial review of the Wallace model fit, we observed the 3.4 correction factor from Wallace (2023) Eq (7) (calculated from PA data from California) substantially underestimated corrected Keene PM_2.5_ concentrations compared to all other models. Therefore, we developed a local correction factor for each heating season in consideration of the specific woodsmoke source in the Keene area and after email discussion with the author (Wallace, personal communication).

Calculating and reporting only one *NMBE* per correction model may not properly demonstrate its performance because the model can have different bias values at different PM concentration ranges. This is an important consideration in exposure assessment and health impact analysis. Therefore, in our comparisons, we assessed the hourly data according to the EPA Air Quality Index (AQI) breakpoints for PM (0 to 9, 9.1 to 35.4, and over 35.5 µg/m^3^) to test how the models performed in each concentration bracket.

Use of the EPA correction model to correct the raw PM_CF_1 data comes with some caveats for epidemiological research. In our study, the EPA model overestimated PM_2.5_ by 13% in the lower concentration ranges (< 9 µg/m^3^ ) and underestimated PM_2.5_ concentrations that were greater than 9 µg/m^3^ by up to 4%. Additionally, the EPA model had higher overall *NRMSE* compared to the local data derived models (Table S1). On balance, the EPA model may be the best selection where personnel resources or the collocated dataset ranges are limited. For epidemiological research, we recommend developing and testing local data models (from sensors collocated near FRM/FEM monitors) as PM composition may vary by region (woodsmoke versus traffic-based PM). Our study was also one of few studies outside of the Wallace research group to apply the Wallace model, which has the benefit of bypassing the proprietary Plantower PM_2.5_ algorithm that calculates the mass concentration data for the PM_CF_1 channel. The Wallace model performed very well in the low (0-9 µg/m^3^) and moderate (9.1 to 35.4 µg/m^3^) concentration ranges in our study with respect to *NMBE* (3.9 % and 2.5%, respectively Table 1). We recommend conducting continued research applying this particle number concentration model to increase the database of case studies in other regions of the country.

In our work, except for the first heating season, we located two PAs next to the reference monitor. In 2019/2020, a second PA, KSC-11, was also located near the BAM (next to KSC-23) the following two heating seasons. We suspect this KSC-11 might have experienced additional unknown problems specific to this sensor. In the diurnal plots in 2019/2020 and 2020/2021, the KSC-11 PA did not track the BAM in the late evening hours and consistently underestimated the BAM concentrations in 2020/2021 (Figure S11). The other PA (KSC-23) dipped under the BAM for late afternoon hours (3 pm to 5 pm) during 2020-2021 (year 3 of collocation).

Additional interunit analyses comparing collocated KSC-23 vs. KSC-11 and collocated KSC-04 vs. KSC-09 showed excellent agreement (Figure S10). KSC-23 vs. KSC-11 in the 2018/2019 season had the strongest correlation (*r* = 0.98, *r*^2^=0.96, *RMSE* = 1.84) compared to the 2019/2021 season (*r*= 0.95, *r*^2^=0.91, *RMSE* = 2.10). This is another line of evidence along with the time series analysis demonstrating KSC-11’s lower performance metrics compared to KSC-23 over time. We observed the best precision between collocated PAs in the 2021/2022 plots for KSC-04 vs. KSC-09 where *r*= 0.99, *r*^2^=0.99 and *RMSE* = 0.94. In general, the precision between the PA units was excellent, which was also supported by the QA/QC data cleaning results.

# Works Cited

Ardon-Dryer, K., Dryer, Y., Williams, J. N., & Moghimi, N. (2020). Measurements of PM_2.5_ with PurpleAir under atmospheric conditions. *Atmospheric Measurement Techniques*, *13*(10), 5441–5458. <https://doi.org/10.5194/amt-13-5441-2020>

Barkjohn, K. K., Gantt, B., & Clements, A. L. (2021). Development and application of a United States-wide correction for PM_2.5_ data collected with the PurpleAir sensor. *Atmospheric Measurement Techniques*, *14*(6), 4617–4637. <https://doi.org/10.5194/amt-14-4617-2021>

Connolly, R. E., Yu, Q., Wang, Z., Chen, Y.-H., Liu, J. Z., Collier-Oxandale, A., Papapostolou, V., Polidori, A., & Zhu, Y. (2022). Long-term evaluation of a low-cost air sensor network for monitoring indoor and outdoor air quality at the community scale. *Science of The Total Environment*, *807*, 150797. <https://doi.org/10.1016/j.scitotenv.2021.150797>

Clark, A., Jones B., Kraft A., and Linera B. (2018). *Beyond the Haze: An Assessment of Air Quality in Keene, New Hampshire* Keene State College. Environmental Studies Department. Unpublished.

Kimberling, T. G., and West, K. (2019). *Predicting Air Inversions Using Neighborhood Based Air Monitoring and Drone Technology: Part of a Program to Encourage Voluntary Reduction in Residential Wood Burning to Improve Air Quality in Keene, NH. Environmental Studies Department. Unpublished.*

Matonte, J., Cobb, A., Burdette, E., & Ang, S. (2020). *Cleaner air spaces in Lane County: Policy interventions for mitigating the health impacts of smoke intrusion events*.

New Hampshire Department of Environmental Services. (2010, September). *Keene Woodstove Changeout Campaign*. <https://www.epa.gov/sites/default/files/2015-09/documents/keenefinalreport2011.pdf>

New Hampshire Department of Environmental Services. (2012, August). *New Hampshire Mobile Air Monitoring Special Study on Small Particles, 2010-2011 and 2011-2012 Executive Report*. <https://www.des.nh.gov/sites/g/files/ehbemt341/files/documents/2020-01/mam-executive-report.pdf>

New Hampshire Department of Environmental Services. (2018, March). *State of New Hampshire Air Quality—2017*. <https://www.des.nh.gov/sites/g/files/ehbemt341/files/documents/2020-01/r-ard-17-01.pdf>

New Hampshire Department of Environmental Services. (2021, December). *State of New Hampshire 2020 Air Quality Update*. <https://www.des.nh.gov/sites/g/files/ehbemt341/files/documents/r-ard-21-05.pdf>

New Hampshire Department of Health and Human Services. (2024, July). *New Hampshire Social Vulnerability Index (SVI) Dashboard*. <https://wisdom.dhhs.nh.gov/wisdom/dashboard.html?category=community-health&topic=social-determinants-of-health&subtopic=social-determinants-of-health&indicator=social-vulnerability-index-(svi)>

Nilson, B., Jackson, P. L., Schiller, C. L., & Parsons, M. T. (2022). Development and evaluation of correction models for a low-cost fine particulate matter monitor. *Atmospheric Measurement Techniques*, *15*(11), 3315–3328. <https://doi.org/10.5194/amt-15-3315-2022>

Robinson, D. L. (2020). Accurate, Low Cost PM2.5 Measurements Demonstrate the Large Spatial Variation in Wood Smoke Pollution in Regional Australia and Improve Modeling and Estimates of Health Costs. *Atmosphere*, *11*(8). <https://doi.org/10.3390/atmos11080856>

Wallace, L. (2023). Cracking the code—Matching a proprietary algorithm for a low-cost sensor measuring PM1 and PM2. 5. *Science of The Total Environment*, *893*, 164874. <https://doi.org/10.1016/j.scitotenv.2023.164874>

Wallace, L., Bi, J., Ott, W. R., Sarnat, J., & Liu, Y. (2021). Calibration of low-cost PurpleAir outdoor monitors using an improved method of calculating PM2. 5. *Atmospheric Environment*, *256*, 118432. <https://doi.org/10.1016/j.atmosenv.2021.118432>
